# Ericaceous vegetation of the Bale Mountains of Ethiopia will prevail in the face of climate change

**DOI:** 10.1038/s41598-022-05846-z

**Published:** 2022-02-03

**Authors:** Yohannes O. Kidane, Samuel Hoffmann, Anja Jaeschke, Mirela Beloiu, Carl Beierkuhnlein

**Affiliations:** 1 Department of Biogeography, University of Bayreuth, Bayreuth, Germany; 2 Bayreuth Center of Ecology and Environmental Research (BayCEER), Bayreuth, Germany; 3 Geographical Institute Bayreuth (GIB), Bayreuth, Germany; 4 Present Address: Department of Environmental System Sciences, Institute of Terrestrial Ecosystems, ETH Zurich, Zurich, Switzerland

**Keywords:** Ecology, Environmental sciences

## Abstract

Climate change impacts the structure, functioning, and distribution of species and ecosystems. It will shift ecosystem boundaries, potentially affecting vulnerable ecosystems, such as tropical Africa's high mountain ecosystems, i.e., afroalpine ecosystems, and their highly susceptible uniquely adapted species. However, ecosystems along these mountains are not expected to respond similarly to the change. The ericaceous woody vegetation, located between the low-elevation broadleaf forests and high-elevation afroalpine vegetation, are anticipated to be affected differently. We hypothesize that projected climate change will result in an upward expansion and increasing dominance of ericaceous vegetation, which will negatively impact the endemic rich afroalpine ecosystems of the extensive Sanetti plateau. Hence, we modeled the impact of future climate change on the distribution of ericaceous vegetation and discussed its effect on bordering ecosystems in the Bale Mountains. We applied four familiar correlative modeling approaches: bioclim, domain, generalized linear methods, and support vector machines. We used WorldClim’s bioclimatic variables as environmental predictors and two representative concentration pathways (RCPs) of the IPCC Fifth Assessment Report climate change scenarios, namely RCP4.5 and RCP8.5 for future climate projection. The results indicate increased ericaceous vegetation cover on the midaltitude of northwestern and northern parts of the massif, and the Sanetti plateau. We observed upward range expansion and increase of close ericaceous vegetation in midaltitudes, while receding from the lower range across the massif. Moreover, the current ericaceous vegetation range correlates to the temperature and precipitation trends, reaffirming the critical role of temperature and precipitation in determining species distributions along elevational gradients. The results indicate the high likelihood of considerable changes in this biodiversity hotspot in Eastern Africa.

## Introduction

The recent climate change induced warming is the most pervasive of the various threats to the planet’s biodiversity^1–3^. Therefore, an urgent challenge in biogeography and ecology is determining how species and ecosystems respond to climatic changes^4^. The recently observed climate change across tropical regions is significantly higher than the global average; for example, there are observed temperature increases for the tropical rainforest regions at a mean rate of 0.26 ± 0.05 °C per decade, with an intensification during the El Niño events^5^. Three out of the four of the IPCC Fifth Assessment Report (AR5) Representative Concentration Pathways (RCPs) predicted moderate to severe climate warming throughout the coming century in response to changes in radiative forcing arising from anthropogenic emissions of greenhouse gases and aerosols^6^. Therefore, climate change will likely induce thermal isotherm shifts increasing the risk of disrupting the stability of afroalpine mountain ecosystems and affecting the unique plant diversity. This can lead to unexpected taxonomic and functional reorganization of communities and massive extinctions of endemic species^1,3,7^.

Mountains are complex landforms that uniquely contribute to biodiversity. Hence, they are important in investigating climate change impact on individual species and ecosystems. They modify regional macroclimates and are endowed with complex microclimatic regimes. The steep gradients of temperature and precipitation and topographic complexity within mountains result in many microsites with a range of adjacent thermal niches^8–10^. In general, there are two categories of environmental changes with altitude: those physically tied to meters above sea level (m asl), such as atmospheric pressure, temperature, and clear-sky turbidity; and those that are not generally altitude specific, such as moisture, duration of sunshine, wind speed, season length, soil depth, geology, and even human land use^9^. Here, we modeled the significance of the temperature and moisture-related bioclimatic variables for the diversity and distribution of ericaceous vegetation and implications for the outstanding afroalpine ecosystems.

The spatial isolation of mountains over a long period has supported the evolution of many endemics^11–13^. Tropical alpine ecosystems above the treeline are particularly isolated and consequently host highly adapted endemic species sensitive to climate change^14–18^. Tropical African high mountain ecosystems, i.e., afroalpine ecosystems, occur in isolated patches restricted to peaks of the high volcanic mountains along the Great Rift Valley and Cameroon-Nigeria Mountain ranges between Tropic of Capricorn and Tropic of Cancer^19,20^. These spectacular ecosystems are habitats to unique plants with specific morphological and functional adaptations that exhibit distinctive traits and distinctive adaptation to diurnal freeze–thaw cycles^21,22^. The afroalpine plant species have long life cycles reflected in woody structures, above and belowground longevity, and limited dispersal capacity resulting in inertia at the ecosystem level^17^. Hence, the rapid range shift of species and ecosystems or local adaptation to novel environmental conditions along mountains is likely to happen by plants with broad phenotypic plasticity and higher dispersal ability.

The Bale Mountains form an enormous contagious massif of extensive plateaus above 3400 m asl in Africa, supporting the extensive ericaceous vegetation^23^. The ericaceous vegetation is a vital component at the transition between broadleaf forests and afroalpine vegetation. It has a broad distribution range, high thermal tolerance, dispersal ability (wind dispersal), and adaptation potential. Key members of this vegetation type may outcompete some exclusively afroalpine plants in case of upward shift^19^. One-third of the afroalpine flora is solely limited to the alpine zone. At the same time, several members of this group of plants have a broader range of distribution that extends to lower vegetation belts, such as the ericaceous vegetation^24^. The term “ericaceous” describes a plant functional type with needle-leaved foliage and a taxonomic group of plants belonging to the plant family Ericaceae. The genus *Erica* comprises acidophilous woody plants^25,26^. In the Bale Mountains, the “Ericaceous Belt” range extends between 3100 and 4200 m asl and are dominated by *E. arborea* L. and *E. trimera* (Engl.) Beentje ^21,23^ (Hence forth *Erica*). The Ericaceous Belt is a resilient ecosystem that serves as a firetrap that promotes and perpetuates the system’s stability^27^. *Erica* leaf shedding builds good surface fuels relatively quickly and stimulates the Ericaceous Belt's fire risk cycle^26,28–31^. However, the traditional fire management system has maintained biodiversity by creating vegetation mosaics, with young, non-flammable stands acting as fuel breaks^29,30^. Besides, soft *Erica* shoots are often grazed and browsed by domestic stock, and the fire-killed *Erica* stumps are collected for firewood by locals.

Most recent SDMs focus on extinction risks of species or groups rare and under threat of extinction, keystone species, or functional types^32^. Little is known about the potential range retraction, expansion, or extinction of the widespread species of the remote landscape such as *Erica*, which are critically important to local ecosystems and biodiversity conservation management. Even slight declines in such species can significantly affect ecosystem structure, function, and services^33^. To date, the extent of *Erica*’s current distribution range and suitable habitat, the impacts of projected climate change in determining its distribution, and the main bioclimatic factors that control its expansion and distribution are not well studied in the Bale Mountains. Relatively, the role of fire, land use, plant diversity distribution, and herbivory as central players in *Erica* dynamics are more researched [e.g., ^22,23,26–31,34–39^].

In addition, the Bale Mountain massif and surrounding lowlands, similar to other parts of East Africa^40^, are inhabited by hundreds of thousands of poor small-scale subsistence farmers. Nsengiyumva^41^ reviewed the last 60 years' anthropogenic temperature increase across eastern tropical Africa and estimated it has increased from 0.278 to 0.72 °C per decade. IPCC^42^ estimated approximately 1.0 °C of global average above pre-industrial levels, likely ranging from 0.8 to 1.2 °C. At the global scale, warming is likely to reach 1.5 °C between 2030 and 2052 if it continues to increase at the current rate^42^. Hence, information from reliable species distribution models (SDMs) enables science to build a basic understanding of ecosystems response and policy to plan reliable mitigation and adaptation measures.

SDMs are especially suited to assess species and ecosystem status overbroad, remote, and inaccessible areas such as mountain ecosystems^7^. Therefore, they are critical to fundamental and applied research in biogeography^43^. We applied SDMs to predict projected *Erica* distribution using WorldClim’s^44^ bioclimatic variables as environmental predictors and two representative concentration pathways (RCPs) of IPCC Fifth Assessment Report (AR5) climate change scenarios, namely RCP4.5 and RCP8.5 for future climate projection. RCP4.5 is the intermediate scenario with emissions peak around 2040, and RCP8.5 is a projection with very high Green House Gas (GHG) emissions that assume emissions continue to rise throughout the twenty-first century. Scenario selection was based on the notion that climate change is happening and partial damage has already occurred. The emission will persist through the first half of the twenty-first century and is anticipated to continue to cause changes in the climate, biological and other systems^42^.

Considering the severity of the anticipated climate change, we asked whether the future environmental conditions would favor further expansion and dominance of plants with a broader habitat range, such as the ericaceous vegetation. We hypothesize that projected climate change will result in an upward expansion and increasing dominance of ericaceous vegetation, which will negatively impact the endemic rich afroalpine ecosystems of the extensive Sanetti plateau. We further hypothesize that due to its broad phenotypic plasticity, *Erica* will respond to the changes and prevail in the area of its potential suitable habitat.

In general, the role of climate change in determining *Erica’s* distribution and its implication to associated alpine and subalpine flora is lacking. Understanding its current and future distribution and ecological range in the face of climate change contributes to biodiversity conservation management planning, and the development of sound climate change adaptation and mitigation strategies and local management strategies. Hence, this research aims to model the current *Erica* distribution range under current bioclimatic conditions and projected climate change. Specifically, the goals are to model the current distribution range of *Erica*, identify the main bioclimatic variables that control Erica’s distribution, model its future potential distribution range*,* and discuss the implication for the afroalpine vegetation.

## Materials and methods

### Study area

#### Location and geology

**Figure 1:**
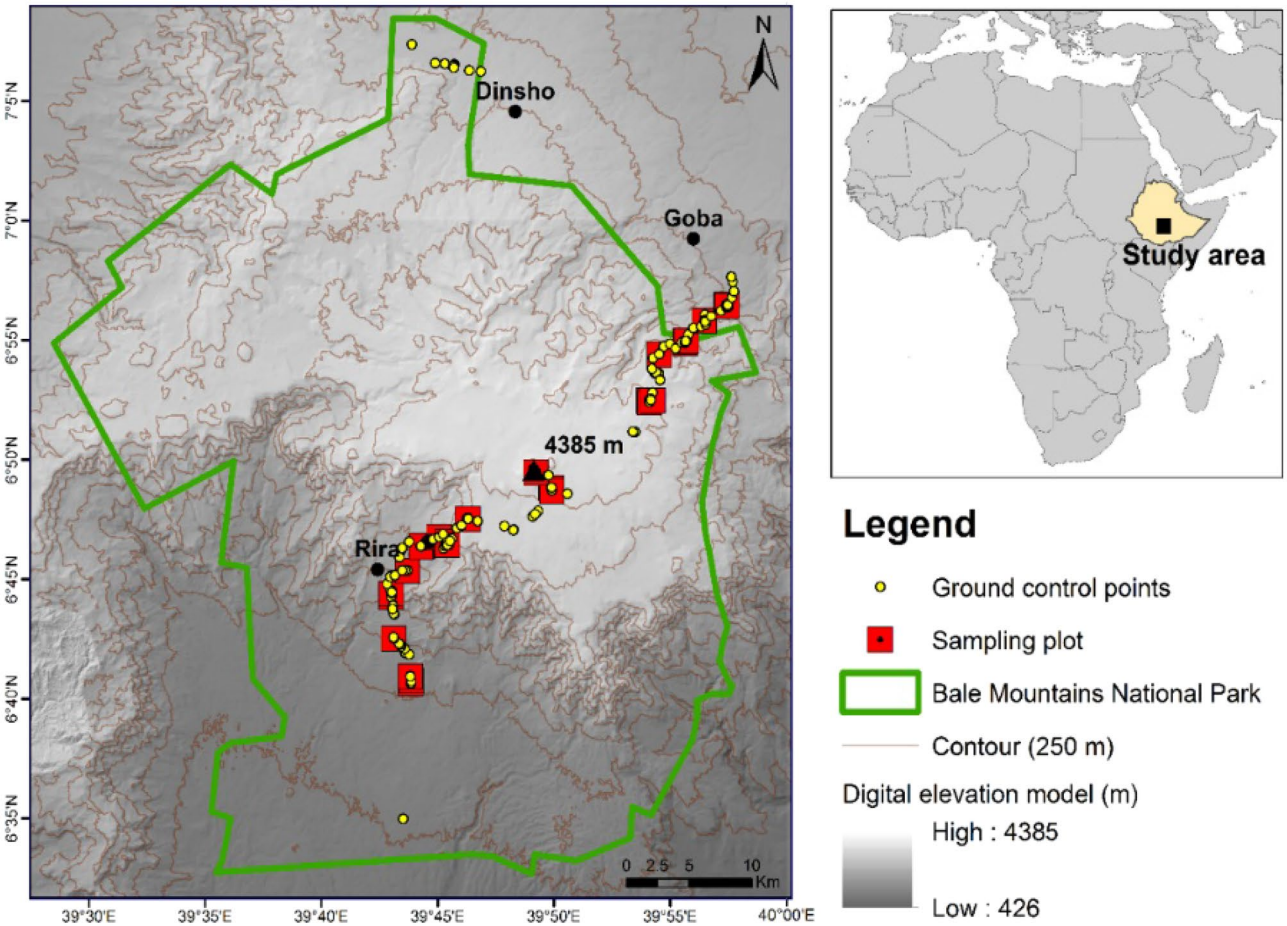
Map of the study area, including the transect sampling plots' location, ground controlling points, and the Bale Mountain National Park (BMNP) boundaries (Prepared by: Mirela Beloiu and Yohannes O Kidane using QGIS 3.4.^52^ and Digital Elevation Model acquired from United States Geological Survey (USGS)^51^.

The study was carried out in the Bale Mountains of Southeastern Ethiopian highlands focusing on the ericaceous vegetation of the massif within an area of geographic extent 39° 25′ E, 40° 00′ E and 06° 25′ S, 07° 10′ N (Fig. 1). The massif is home to the most extensive afroalpine ecosystems in Africa. They were once extensively glaciated, and the entire climate was much colder and drier during the glacial periods of the Pleistocene, which shaped their recent geomorphology^36,45^. The mountains are fragmented by numerous volcanic plugs, peaks, alpine lakes, and rushing mountain streams that descend into deep rocky gorges. The mountains, especially the afroalpine proper and ericaceous Belt, were pushed down by ∼1000 m and covered larger areas than today during a long period in the Pleistocene^27,36,46,47^. These species assemblages are spatially condensed today.

#### Climate

The Bale Mountains are located at the convergence of the wet East African and dry northeast African mountains of southeast Ethiopia. Rain comes to the mountains from two different sources, the equatorial westerlies (rainfall pattern influenced by the Intertropical Convergence Zone (ITCZ)) and the Indian Ocean Monsoon^37^. The south and southwest slopes are more humid with a subtropical climate and high annual rainfall up to 1500 mm/year. The north and northeastern parts experience an annual rainfall from 800 to 1100 mm and a wet season from June to September. Along Harenna escarpments, precipitation increases to around 3800 m asl, then decreases toward the summits^34^.

The afroalpine region above 3400 m asl to the summit^23^ is often covered with clouds and gains less precipitation than the Afromontane range 1500 m asl to around 3250 m asl^24,38^. In general, the afroalpine climate is cold and wet, except in the short dry season, usually lasting from December to January or into March–April during drought years^23^. Unlike the wet season, the dry season has relatively higher daytime maximum temperatures and lower nighttime minimum temperatures at the tropical alpine zone^20^. Diurnal freeze–thaw cycles, yet slight seasonal variations in temperature, are typical in the alpine zone^21,22^. This "summer every day, winter every night" pattern is characteristic of tropical alpine areas^20,48^. Hillman^34^ recorded an extreme diurnal temperature range of about 40 °C (− 15 to + 26 °C) during the dry season.

#### The Ericaceous Belt

The Bale Mountains exhibit a steep gradient of ecological zones ranging from tropical rainforests to afroalpine vegetation^24,49^. The area above the upper montane forest, above 3100 m asl, is dominated by the two closely related *Erica species,* *E. arborea* L. and *E. trimera (Engl.)* Beentje otherwise known as “the Ericaceous Belt” ^21,23^. Both species have similar morphology, distribution, and habitat ecology, making it difficult to distinguish the two species in the field^28^. *E. arborea* is widely distributed in Africa, the Middle East, and Europe. At the same time, *E. trimera* is endemic to the afroalpine mountains and occurs in several mountain systems of East Africa and the Ethiopian Highlands^23^. *E. trimera* tends to dominate at higher elevations between 3700 m asl to 4150 m asl, while *E. arborea* has a broader distribution range that extends between 3100 and 4200 m asl^23,50^, usually extending 1000–1100 m vertical distance.

**Figure 2:**
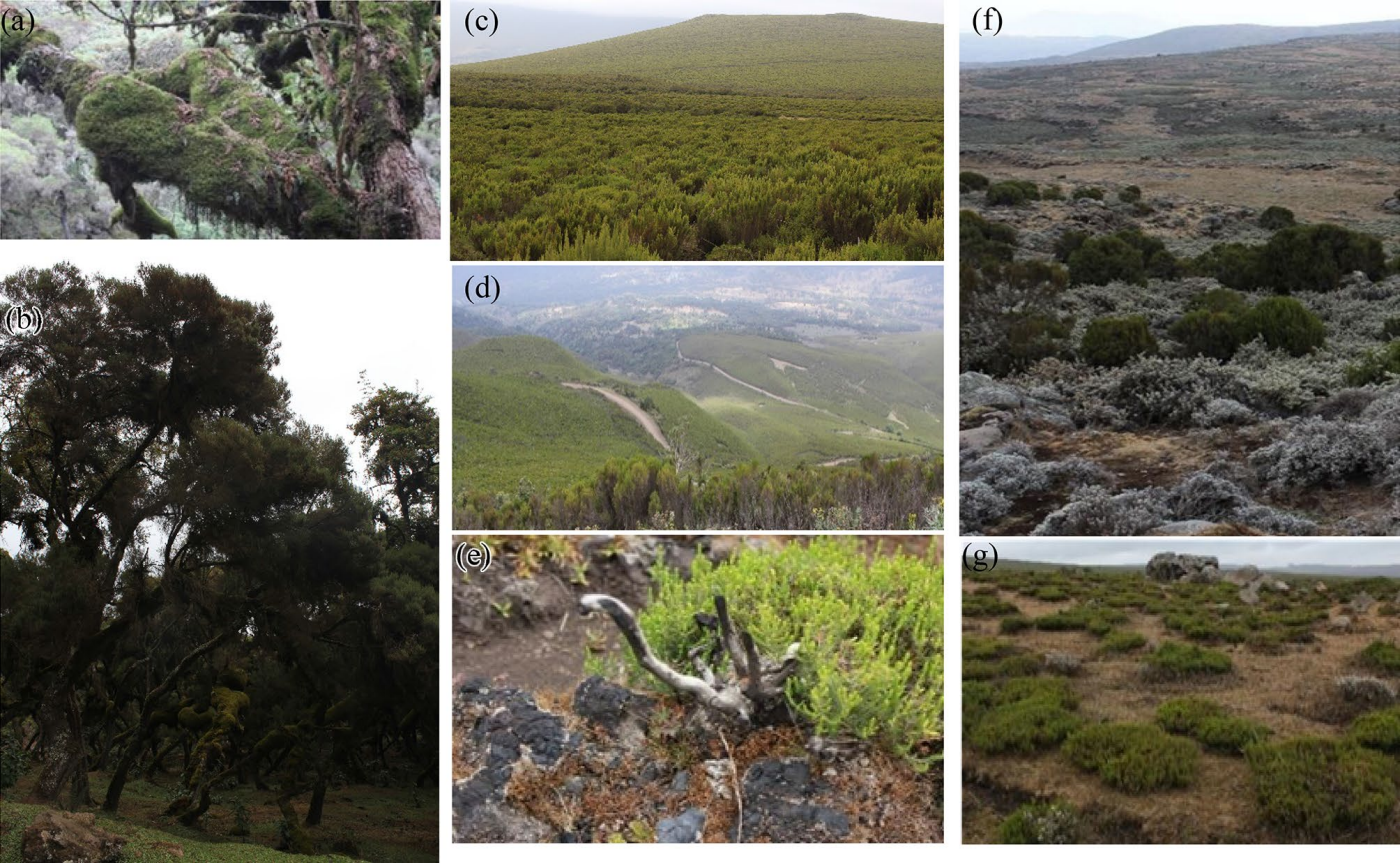
*Erica* succession and distribution patterns along the Bale Mountains. (**a**,**b**) Old Erica tree stand up to 12 m high with thick *Breutelia spp.* Moss on the stem, (**c**,**d**) Ericaceous Belt: young Erica brush 1–5 years old (1–3 m high), (**e**) *Erica* lignotubers regeneration after a fire event, and (**f**,**g**) Isolated *Erica* groves at the *Erica a*froalpine grassland ecotone mainly dominated by isolated *Erica* shrubs and different grasses.

*Erica* displays different phenotypes across elevational gradients (Fig. 2), mainly driven by environmental factors such as temperature and moisture^23,28,35^. It appears as evergreen dwarf shrubs of a few centimeters high to trees up to 12 m^23,28^. This older *Erica f*orest has dense multi-stem trees covered with prominent epiphytes such as mosses and ferns. Above the old growth is the mid-altitude of *Erica* distribution, the dense shrubland covered with shrubs of approximately 0.5–3 m. Here, in between the resprouting *Erica* shrubs are distinct grasses and herbs growing. Finally, it occurs as a few centimeter-high shrubs at a higher elevation.

The Sanetti Plateau covers areas that start at above 3400 m asl, the alpine treeline, to the summits. The slopes are covered with lower-statured heather that merge and difusses into afroalpine vegetation. Here, *Erica* is kept in a shrubby state up to around 4200 m asl through repeated burning, freezing temperature, rodent disturbance, and grazing^23,28^.

On the Sanetti Plateau, the plants are composed of low-statured, Tussock grass, perennial life forms, and giant rosettes, e.g., low-stature woody shrubs, herbaceous forbs, graminoids, cushions with the relative abundance of each related to their location within the massif and local microclimate. Plants like *Dipsacus pinnatifidus* Steud. ex A. Rich*., Eriocaulon schimperi* Körn. ex Ruhland, *Carex monostachya* A. Rich., *Helichrysum splendens* Sims*, Helichrysum citrispinum* Delile.*, Helichrysum cymosum* (L.) D. DON., *Geranium kilimandscharicum* Engl.*, Alchemilla abyssinica* Fresen., *Alchemilla rothii* Oliv., *Artemisia afra* Jacq. ex Willd. and *Polygonum afromontanum* Greenway are common. The spectacular uniquely adopted giant rosette plants*,* such as *Lobelia scebelii* Chiov., *Lobelia giberroa* Hemsl, and *Scenecio spp.,* are the most prominent plant. Other significant plants such as Tussock grass-like *Pentaschistis minor* Ballard and Hubbard and *Festuca abyssinica* Hochst are common on the afroalpine plateau.

### Data collection

#### Ground controlling points collection and data preparation

**Figure 3:**
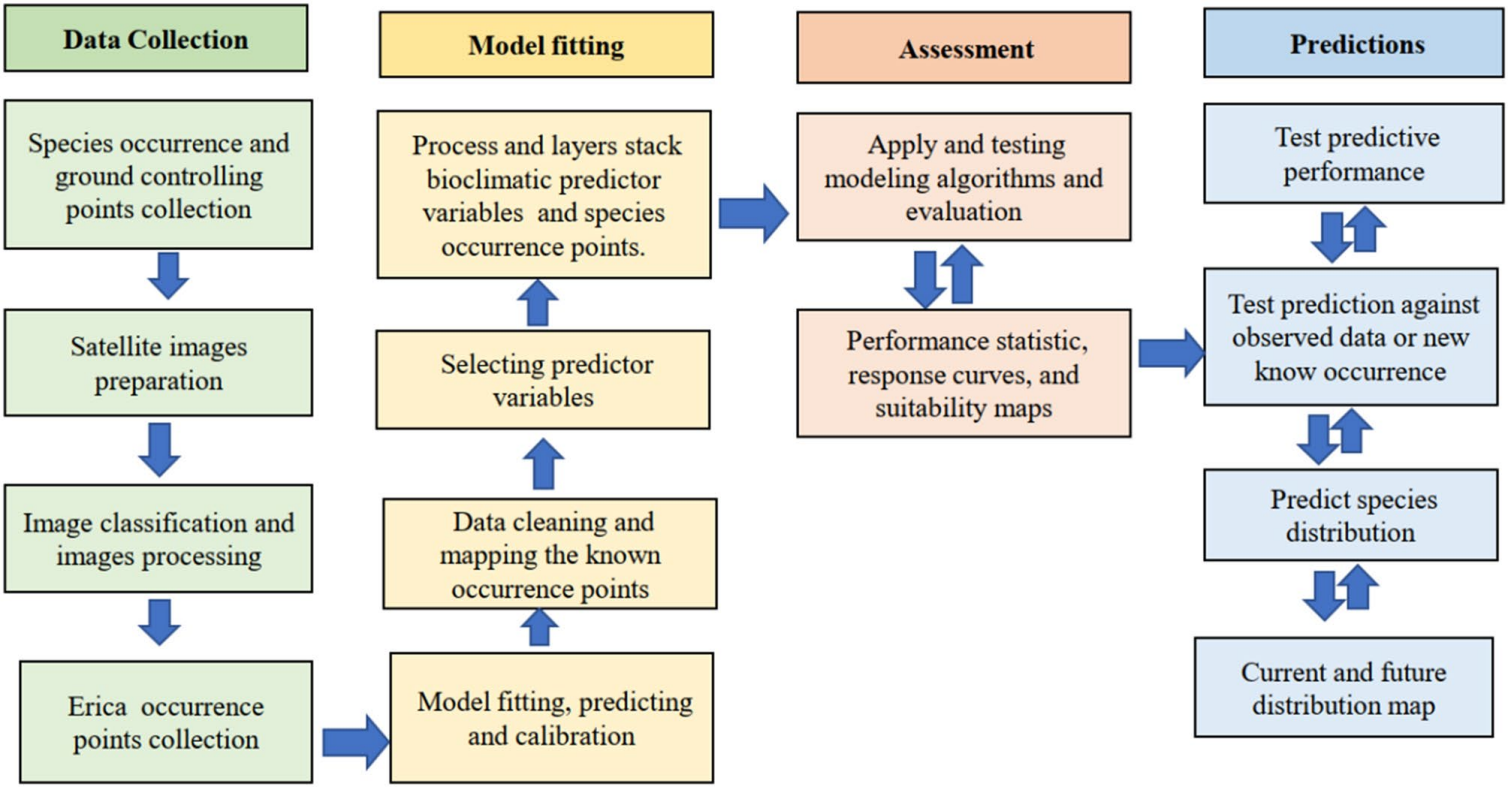
Modeling flow diagram detailing the main data collection and preparation steps, modeling fitting, assessment and evaluation, and prediction and validating steps.

Landsat TM + 8 remote sensing images 30 m × 30 m resolution from March 8, 2017, paths 167 and 168, and raw 55 and 56 were acquired from the United States Geological Survey homepage^51^. The data preparation and modeling steps are described in Fig. 3. Ground control points were collected from the vegetation sampling plots and GPS recordings collected across the Bale massif during our field visit in March 2011. We used the ground control points to identify the cover classes and classification accuracy assessment. Besides, high-resolution Google Earth images and pictures (https://earth.google.com) were used to crosscheck any recent vegetation cover changes during the image classification. The altitude and coordinates of each sampling plot were recorded using Garmin GPS 3.1.

#### Image classification and occurrence data collection

Georeferencing, image classification, occurrence points extraction, and coordinates crosschecking were carried out using QGIS 3.4.^52^. We applied the QGIS Semi-Automatic Classification image processing approach. The object based supervised maximum likelihood algorithm was used to classify the images. In such an approach, the analyst defines areas where the landcover is known, predefines the Land Use Land Cover (LULC) types and the number of classes based on selected parameters^53,54^. This approach enhances the delineation objectivity, interpretation repeatability, and processing efficiency^55^.

We classified the images based on the area's known eight major vegetation classes^24^. The classification accuracy was evaluated using the kappa coefficient^56^. We achieved an overall accuracy of 93%. Usually, the image classification Kappa coefficient and overall accuracy above 73% are acceptable and required^53^.

After the final classification, the landcover classes other than *Erica* were masked, and occurrence points were extracted. More than 3220 *Erica* occurrence points were collected from the final known *Erica* cover class. The modeling, including further data preparation, cleaning, and calibration were carried out using R version 3.6.0^57^ based on the SDMs steps described in Hijmans and Elith (2017) (See supplementary material). *Erica's* occurrence duplicate points were removed from the database during the modeling proceedings. Error-free and adequately representing occurrence points were compiled and used for modeling.

#### Predictor variables selection and preparation

Model outputs are primarily driven by choice of predictor variables fitted into the models and the type and level of adjustment between the response and predictor variables^43,58,59^. We used the bioclimatic variables derived from the monthly temperature and rainfall values provided by WorldClim version 1.4^44^ as our main environmental predictors. We downloaded the historical (current) climate data for 1970–2000 and future 2050s (projected climate data for 2046–2065) and 2070s (projected climate data for 2081–2100) of 19 bioclimatic predictors variables at 30 arc sec (~ 1 km^60^) spatial resolution.

We selected two IPCC Fifth Assessment Report (AR5) climate change scenarios, Representative Concentration Pathways (RCPs)^42^ of the Coupled Model Intercomparison Project Phase 5 (CMIP5), which were derived from the output of coupled atmosphere–ocean general circulation models (AOGCMs)^61^. We used RCP4.5 (2050s) and RCP8.5 (2070s). RCP4.5 is an intermediate scenario or likelihood that climate change will be constrained to 2–3 °C above pre-industrial levels and RCP8.5 as a high warming scenario, with an average temperature increase of 3.7 °C (2.6–4.8) because it assumes emissions continue to rise throughout the twenty-first century.

Species occurrence data and the types of environments in which species prevail are important because SDMs are sensitive to sample size and biases in data distribution^43^. We tested the 19 bioclimatic variables for collinearity using Variance Inflation Factor (VIF), VIFcor functions, in R within the USDM package^59^. VIFcor measures the severity of multicollinearity in regression analysis and is a pairwise correlation that excludes the highly correlated variables stepwise. We applied the default threshold of 0.9, and variables above the threshold were excluded. Consequently, ten predictor variables were identified as suitable predictors for the *Erica* SDM Table 1.

**Table 1:** The identified ten suitable bioclimatic variables which were used to generate the *Erica* distribution models.

No	Environmental predictor variable	Code
1	Mean Diurnal Range (Mean of monthly (max temp–min temp))	Bio2
2	Mean Temperature of Wettest Quarter	Bio8
3	Mean Temperature of Driest Quarter	Bio9
4	Mean Temperature of Warmest Quarter	Bio10
5	Mean Temperature of Coldest Quarter	Bio11
6	Precipitation of Wettest Month	Bio13
7	Precipitation of Driest Month	Bio14
8	Precipitation Seasonality (Coefficient of Variation)	Bio15
9	Precipitation of Warmest Quarter	Bio18
10	Precipitation of Coldest Quarter	Bio19

### Data analysis

#### Model fitting, prediction, and evaluation

**Figure 4:**
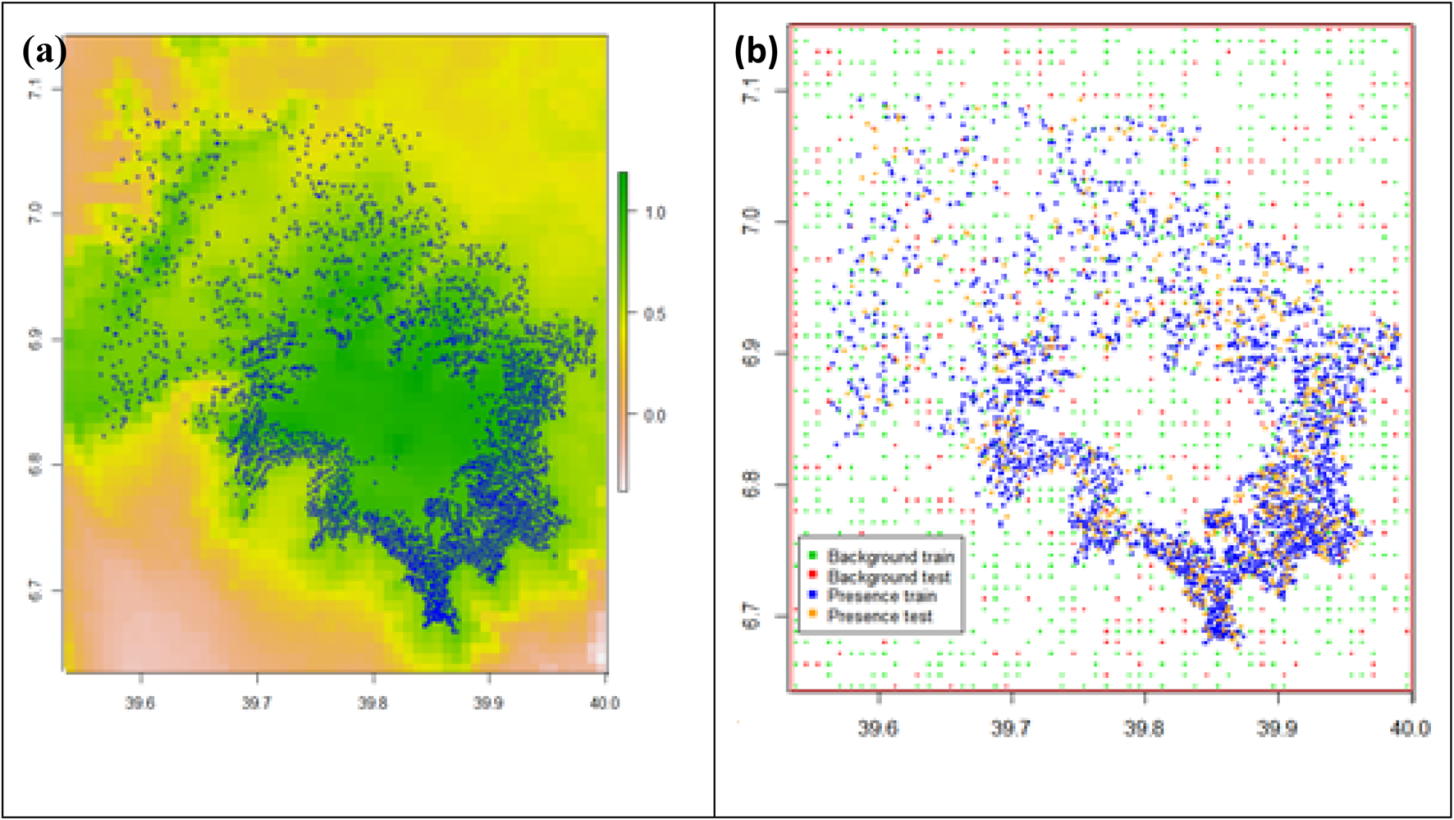
(**a**) Background: Layer stacked environmental predictors variables and sampled *Erica* (Blue circles) occurrence points, (**b**) Model fitting map with background train (green), background test (red), presence train (blue), and presence test (orange).

The modeling, including data preparation, cleaning, and calibration, was carried out following the SDMs r-script presented in^62^. The predictor variables were of the same spatial extent, resolution, origin, projection, and organized as raster ".tif” data. They were layer stacked (Fig. 4a). The dependent (predicted values) and independent variables (bioclimatic predictors and *Erica* presence values) were identified, then the models were fitted. Cross-validation was carried out by creating a training and testing data set through random sampling and modeling with the data set of known occurrences (Fig. 4b).

The *Erica* occurrence database, together with ten current and future bioclimatic predictors, were preprocessed with R version 3.6.0, using the packages "dismo” and “raster”. The "dismo" package is used to predict the environmental similarity of any site to that of the locations of known occurrences of a species^63^. The "raster” package provides a spatial (geographic) data structure that divides a region into rectangles called “cells” or “pixels” that can store one or more values for each of these cells^64^. Subsampling within the range of our study area was run to reduce sampling bias and produce more balanced samples for model calibration. The modeling data sets were created through random sampling from a single data set. 1572 (75%) training and 487 (25%) test values were sampled for model evaluation.

Model performance was evaluated by calculating area Under the Curve (AUC). AUC is a measure of rank-correlation commonly used in SDM studies because it is insensitive to species prevalence and does not require a threshold value to convert probabilities to presence-absence^62^. A high AUC indicates sites with high predicted suitability (areas of known presence) in unbiased data, while AUC below the threshold indicates species absence. Elith et al.^65^ in their model comparison, they found the best SDM models for each of their species distribution predictions had an AUC value of > 0.75; our models' AUC value was 0.79.

#### Modeling methods

We selected four widely used correlative modeling methods Bioclim, Domain, Generalized Linear Methods (GLMs), and Support Vector Machines (SVMs), because of their wide use and good predictive performance. These algorithms have proven to perform well for species distribution modeling that uses species occurrence data and bioclimatic variables^65^. They all compute habitat similarity by comparing the values of bioclimatic variables to a percentile distribution of known species occurrence locations. The Bioclim algorithm has been one of the leading SDM packages for many years and remains widely used^66^. The most common climate data source for SDM studies, the WorldClim database, was created using climate interpolation methods developed applying BIOCLIM^66^. The Domain algorithm computes the Gower distance between environmental variables at any location and those at any known species occurrence locations^67^. The algorithm assigns to a place the distance to the closest known species occurrence. Generalized Linear Models (GLMs) are used extensively in species’ distribution modeling because of their solid statistical foundation and ability to model ecological relationships realistically^60^. These models fit parametric terms, usually some combination of linear, quadratic, and/or cubic terms. GLMs are generalizations of ordinary least squares regression. Support Vector Machines (SVMs)^68^ applies a simple linear method to the data but in a high-dimensional feature space that is non-linearly related to the input space.

Finally, we applied model averaging to produce a more reliable model ensemble prediction. Model ensembles are fitted and evaluated to project potential species distributions in space and time^58,59,69^. The use of many models and applying model averaging to reduce reliance on a single model is suggested by many as a sound approach for better predictions [e.g.,^58,62,70^]. Hence, the four individual model predictions were ensembled based on their AUC values, and the mean predicted values were calculated. Furthermore, we calculated the difference between the averaged models of the different time steps, i.e., between current and the 2050s, and current and 2070s.

## Results

The results of each modeling method are presented below. The probabilistic occurrence prediction maps obtained from the model runs, and corresponding presence/absence maps calculated based on each models threshold value are presented. These maps were built based on the most common threshold optimization method, the Cofusion matrix (max TPR (True Positive Rate) + TNR (True Negative Rate)^58,62,65^. It is used to measure the performance of the classification model^58^ (see Supplementary material). Each method has a different threshold depending on the model's algorism. The values above the threshold were marked as species presence, i.e., environmental conditions are optimal that allow Erica to satisfy its minimum requirements to flourish.

### The bioclim predictions

**Figure 5:**
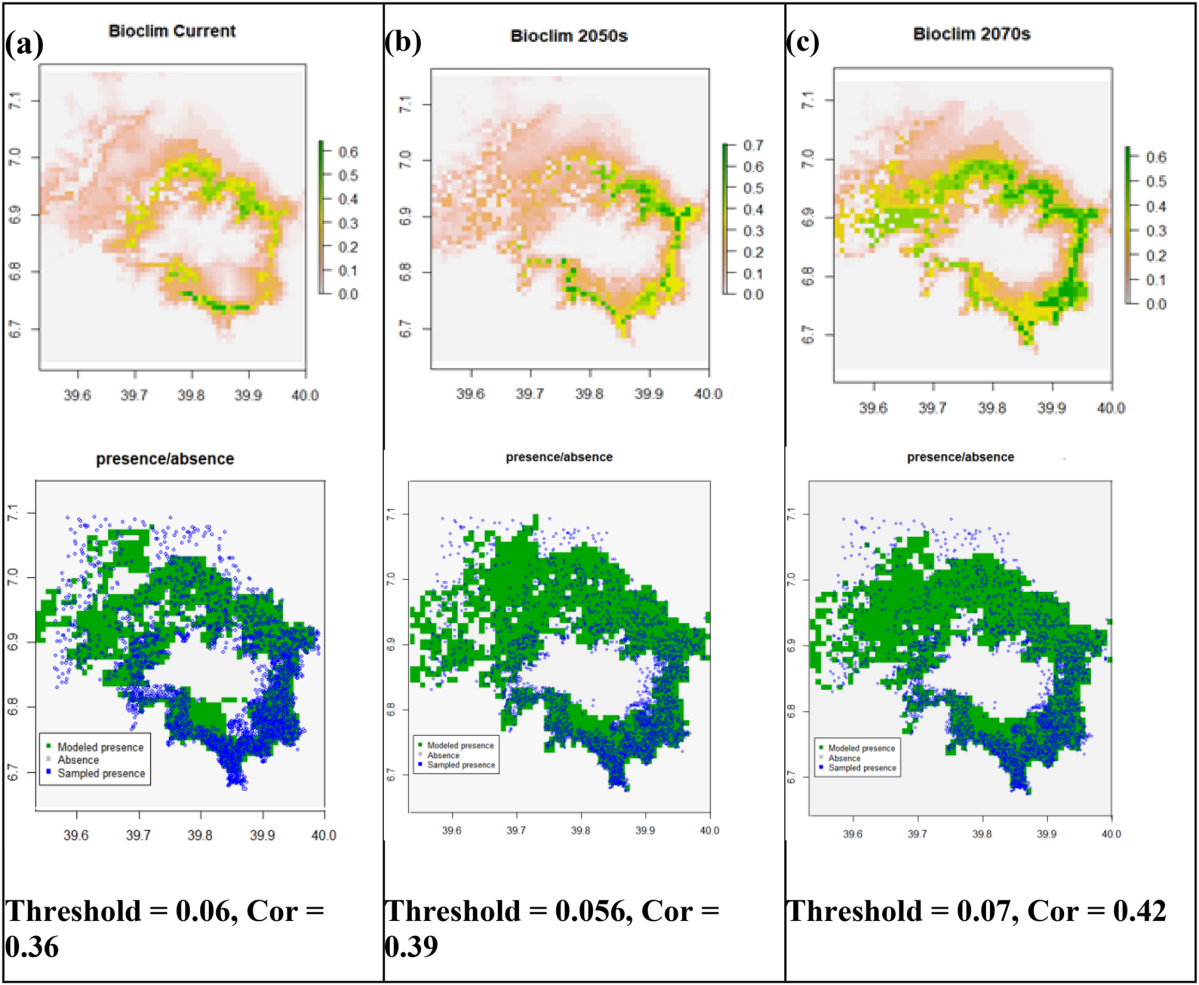
Bioclim’s prediction maps of probability of *Erica* occurrence for current, future 2050s and future 2070s, and their respective presence/absence maps compared with sampled presence sampling points. A gray area represents absolute absence (unsuitable habitat), green to orange indicates modeled presence (suitable habitat), while blue circles represent sampled presence points.

The Bioclim model predicted *Erica's* current and future occurrence ranges with AUC values 0.82, 0.84, and 0.84 for current, the 2050s (RCP4.5), and the 2070s (RCP8.5), respectively (Fig. 5). The probabilistic occurrence prediction maps *Erica’s* presence with a threshold of 0.06, 0.056, and 0.07 for current (Fig. 5a**)**, the 2050s (Fig. 5b**),** and 2070s (Fig. 5c**),** respectively. The future models predicted *Erica’s* persistence within the current range while expanding towards western parts (the Web Valley and the area west of Lencha Ridge). This area is mainly covered with afroalpine *Helichrysum* dwarf shrubs and herbaceous formations. Besides, the future models predicted *Erica's* consolidation within the current range, particularly on the eastern, norther and northeastern parts around Garba Gurecha, while shrinking from all lower altitudes of southwester and western parts.The Bioclim model predicted *Erica’s* current distribution range well across the massif.

### The domain predictions

**Figure 6:**
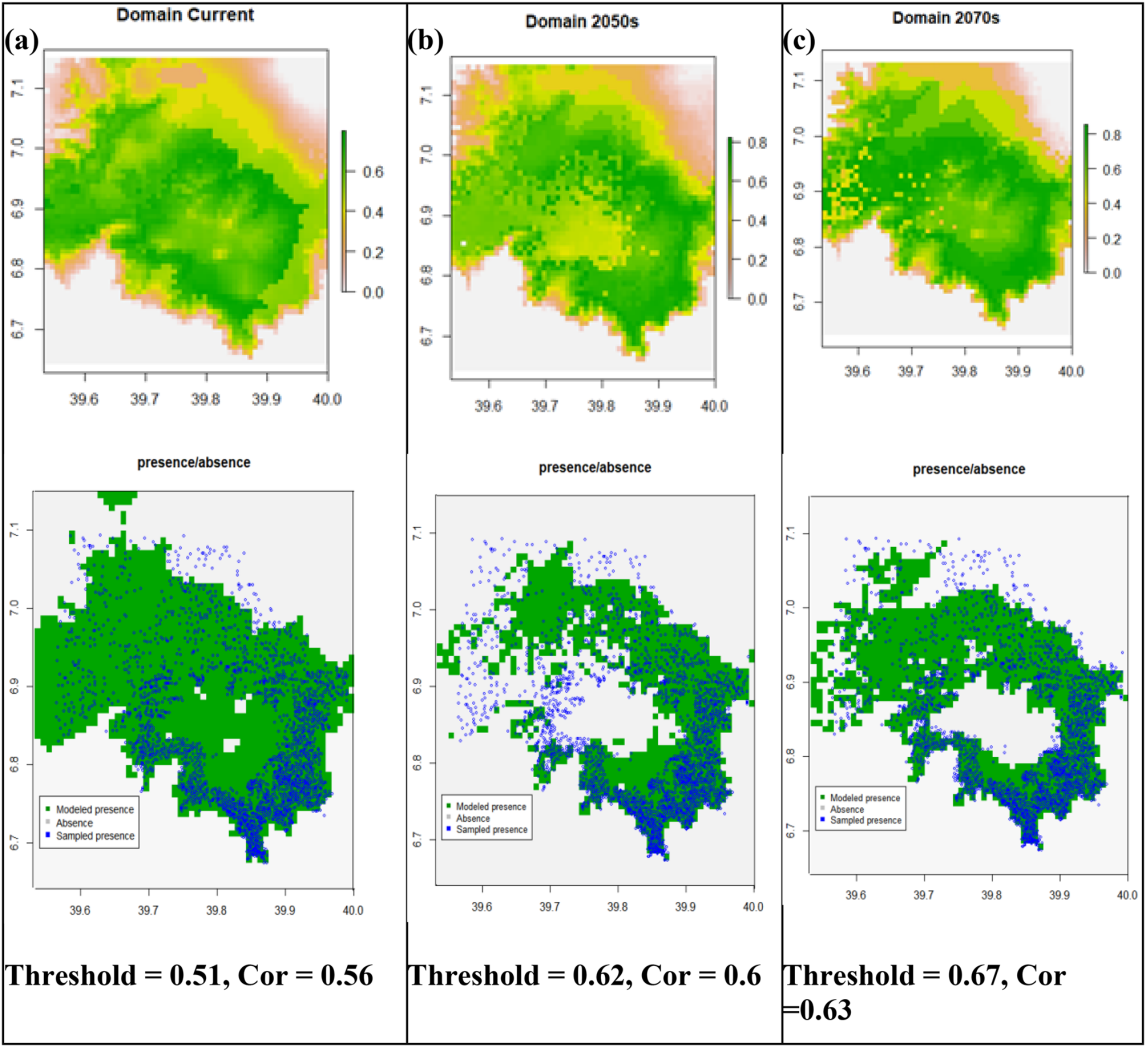
Domain’s prediction maps of probability of *Erica* occurrence for current, future 2050s and future 2070s, and their respective presence/absence maps compared with sampled presence sampling points. A gray area represents absolute absence (unsuitable habitat), green indicates modeled presence (suitable habitat), while blue circles represent sampled presence points.

The Domain model predicted *Erica*'s current and future occurrence ranges with model AUC values 0.81, 0.83, and 0.83 for current, the 2050s (RCP4.5), and the 2070s (RCP8.5), respectively (Fig. 6). The probabilistic occurrence prediction maps with optimization threshold 0.51, 0.62, and 0.67 for current (Fig. 6a**)**, the 2050s (Fig. 6b**)** and 2070s (Fig. 6c**)** respectively. The Domain method, unlike the Bioclim, modeled the afroalpine main habitat, the Sanetti Plateau, as a highly suitable *Erica* habitat, even area contemporarily no inhabited by *Erica*. The future models, however, predicted successive consolidation of *Erica* within the current southeatern eastern and northeaster parts, and retraction from the western and northern during the 2050s, and recolonization of the western and northern parts by 2070s. The future models does not show strong upwards expansion towards the top of the afraolpine plateau, the Sanetti Plateau. They futher indicated habitat simplification and retracting of *Erica* from all its lower ranges on the norther, western and southwestern parts.

### Generalized linear model (GLM)

**Figure 7:**
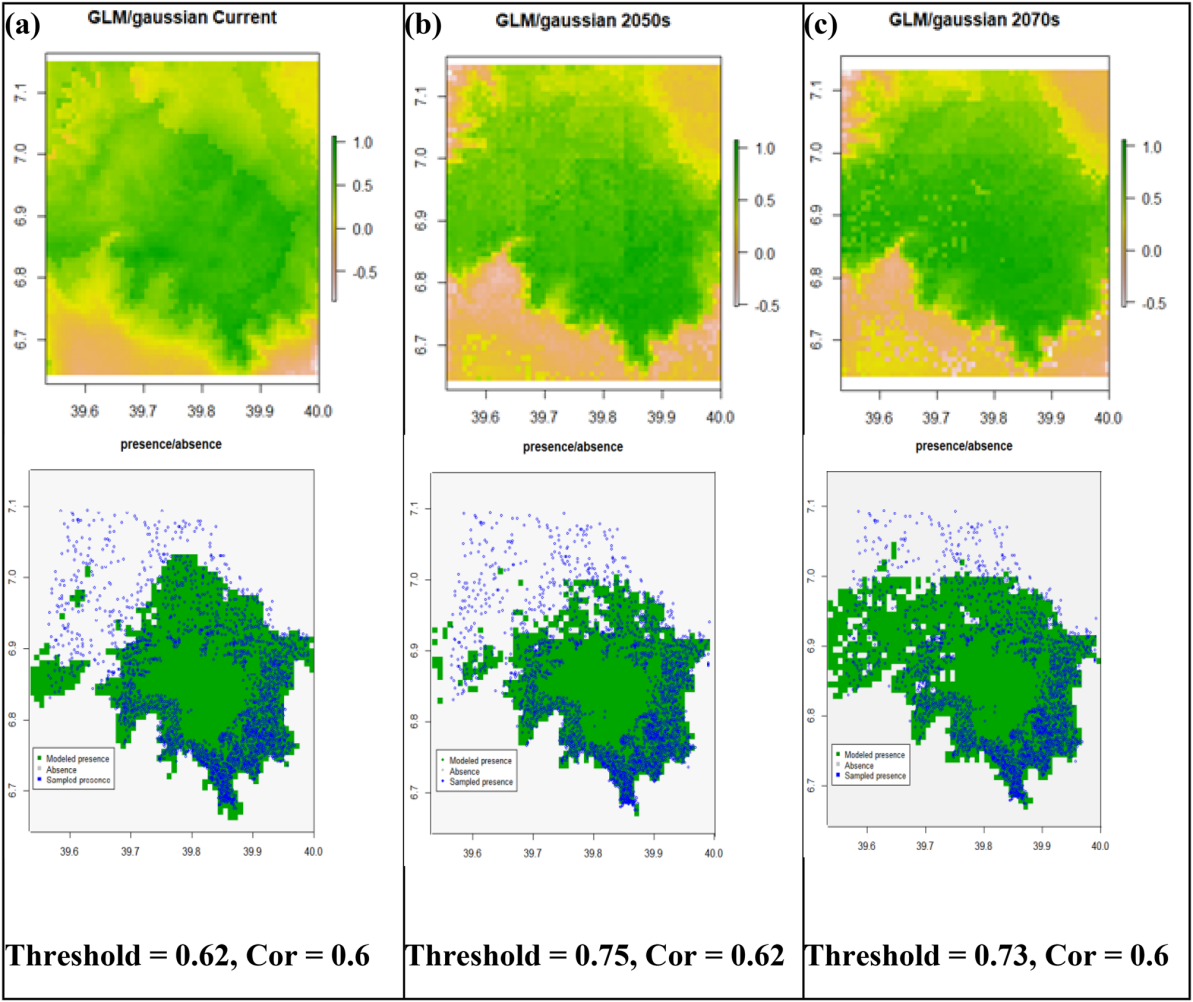
Generalized Linear Model’s (GLM’s) prediction maps of probability of *Erica* occurrence for current, future 2050s and future 2070s, and their respective presence/absence maps compared with sampled presence sampling points. A gray area represents absolute absence (unsuitable habitat), green indicates modeled presence (suitable habitat), while blue circles represent sampled presence points.

The GLM model predicted *Erica*'s current and future occurrence ranges with model AUC values 0.83, 0.86, and 0.81 for current, the 2050s (RCP4.5), and the 2070s (RCP8.5), respectively (Fig. 7). The probabilistic occurrence prediction maps with optimization threshold 0.62, 0.75, and 0.73 for current (Fig. 7a**)**, the 2050s (Fig. 7b**)** and 2070s (Fig. 7c**)** respectively. The future prediction indicated *Erica's* substantial north and northwestern ward expansion with time while losing substantially in all the lower ranges. The GLM model similar to the Domain method modeled the afroalpine main habitat, the Sanetti Plateau, as a highly suitable *Erica* habitat, even area contemporarily no inhabited by *Erica* for all time steps. The current model fail to model some parts of the current Erica habitats, such as the norther, western and southwestern parts of the massif (the Web Valley and the area west of Lencha Ridge). Both future projections indicate a solid midaltitude persistence of *Erica* within its current range, while retreating from all lower ranges of the southern and eastern parts of the massif.

### Support vector machine (SVM)

**Figure 8:**
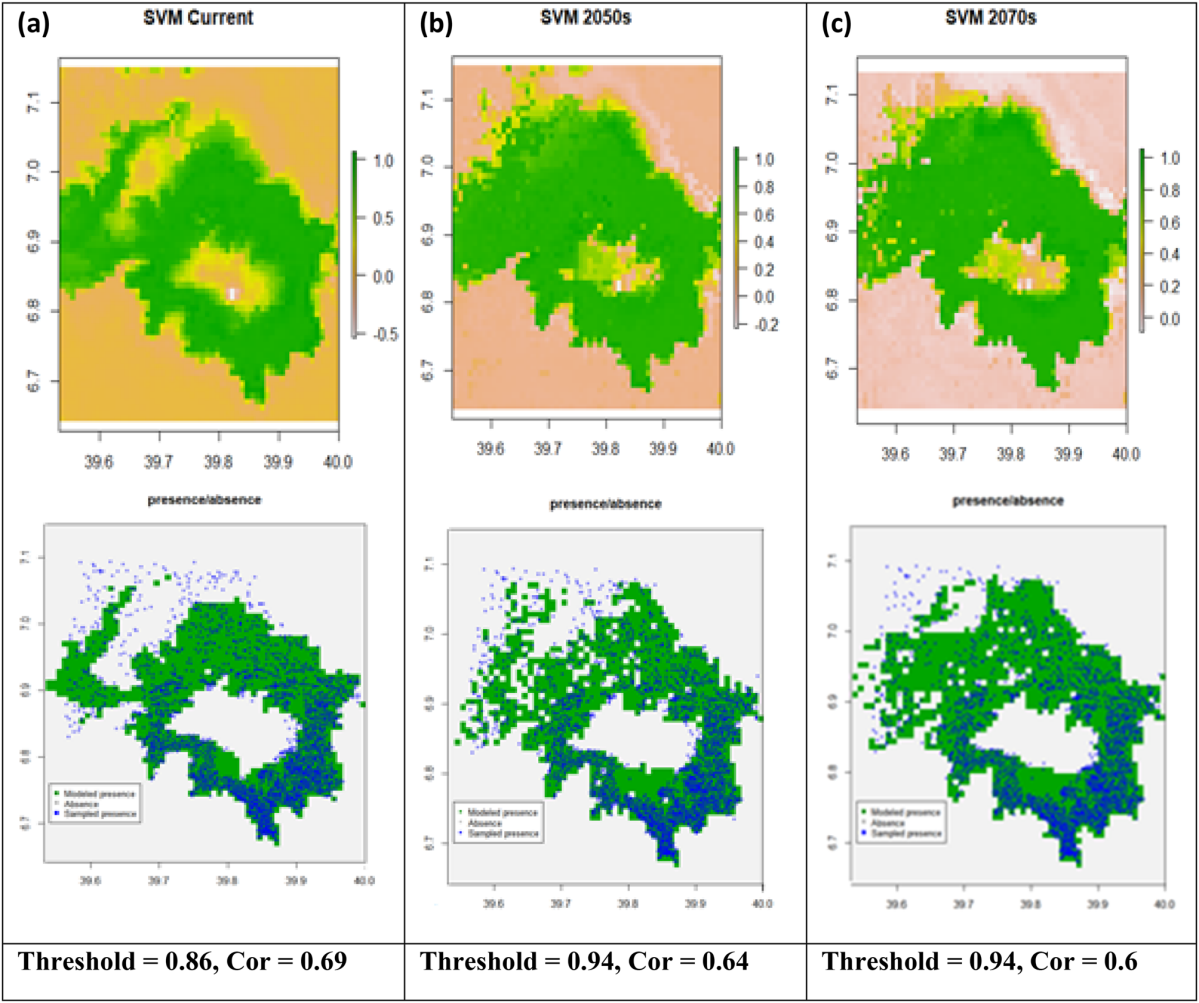
Support Vector Machine’s (SVM’s) prediction maps of probability of *Erica* occurrence for current, future 2050s and future 2070s, and their respective presence/absence maps compared with sampled presence sampling points. A gray area represents absolute absence (unsuitable habitat), green indicates modeled presence (suitable habitat), while blue circles represent sampled presence points.

The SVM model predicted *Erica's* current and future occurrence ranges with model AUC values 0.86, 0.83, and 0.82 for current, the 2050s (RCP4.5), and the 2070s (RCP8.5), respectively (Fig. 8). The probabilistic prediction maps obtained from the model runs and projections were split into binary presence-absence maps. The probabilistic occurrence prediction maps with optimization threshold 0.86, 0.94, and 0.94 for current (Fig. 8a), the 2050s (Fig. 8b) and 2070s (Fig. 8c) respectively. The future predictions indicated a substantial consolidation of *Erica* within its current range with limited simplification on the western part, westward expansion into the current dispersed *Erica* habitat, slight expansion towards the afroalpine range, and pronounced loss on all areas of the massifs lower ranges. Similar to the Bioclim model, SVM predicted *Erica’s* current distribution range well across the massif, except for some the norther and northerwestern parts of the massive.

All four models predicted the loss of suitable habitat at lower ranges, especially at the southwester range, while the substantial gain on the mountain's western, northern, and eastern parts.

### Ensemble model predictions

**Figure 9:**
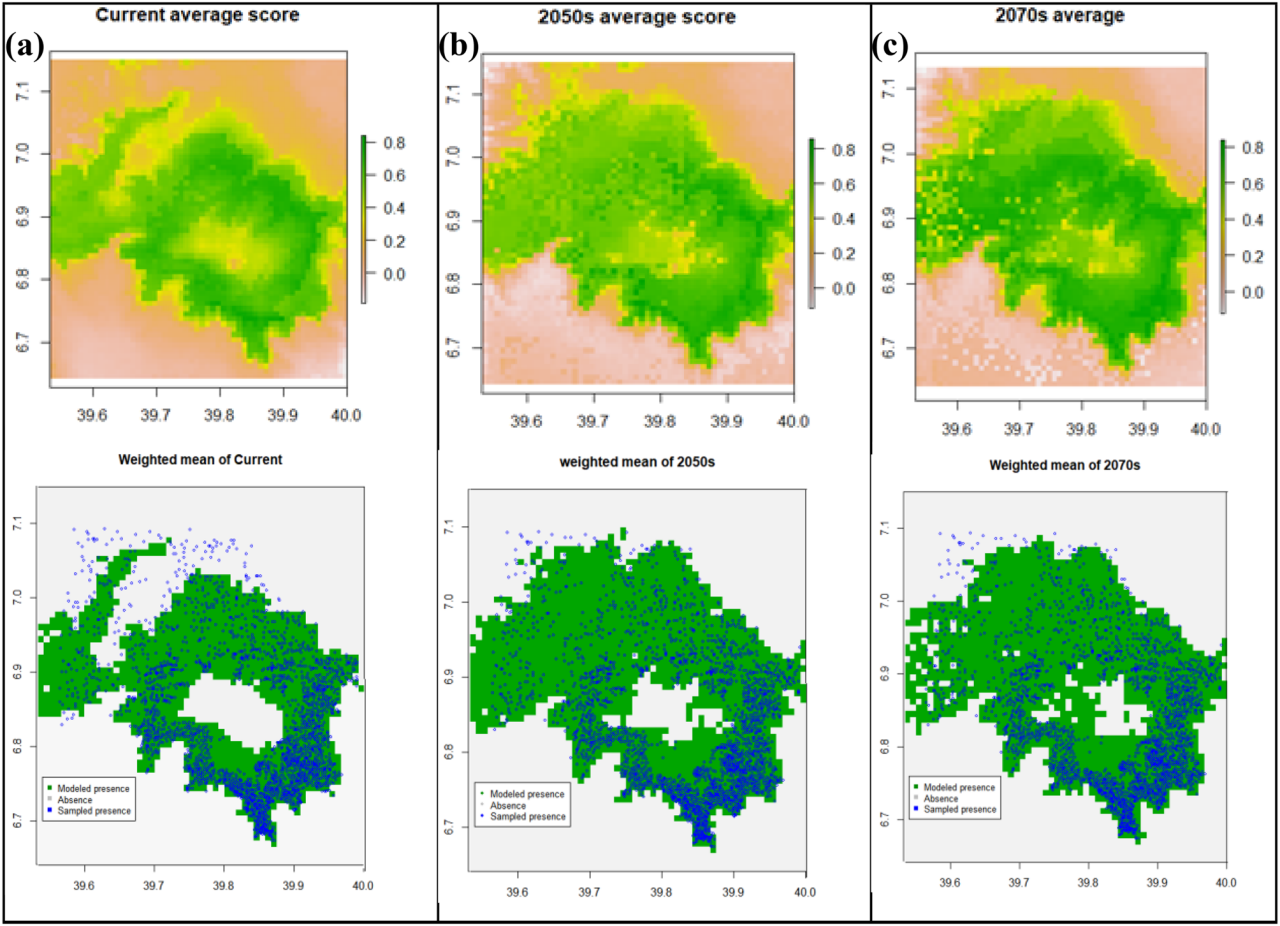
Ensemble four models’ average of the three-time steps (Current, 2050s, and 2070s), and their respective presence/absence maps compared with sampled presence sampling points. A gray area represents absolute absence (unsuitable habitat), green indicates modeled presence (suitable habitat), while blue circles represent sampled presence points.

In order to optimize species distribution predictions and range shift under global change, rather than relying on a single “best” model, some authors [e.g.,^58,62,69,70^] suggested using many models and applying model averaging. The results of our four individual model predictions were weighted by their AUC scores. To create the weights^62^ subtracted 0.5 (the random expectation) and squared the result to give additional weight to higher AUC values (Fig. 9a–c). The probabilistic occurrence prediction maps were split into binary presence-absence maps by averaging the optimization threshold of all four models. Hence, we used a threshold of 0.5.

The averaged model projected habitat loss on all the lower ranges of the current *Erica* distribution range in the future, substantial consolidation of *Erica* on most of its present habitats, significant expansion towards the western, northern, and eastern part of the massif, and the afroalpine plateau, while retracting from the lower ranges of the eastern, southern and northeeatern parts of the plateau. Similar to the Bioclim and SVM modles the models ensseble produce occurrence prediction map that mirros the current range. The ensembled models built with model averaging are promising for modeling species distribution^58,62,69^.

### Change calculation between current and future predictions

Finally, we run a change detection matrix between the averaged models of the different time steps, i.e., between current and the 2050s (Fig. 10.1a–c) and current and 2070s (Fig. 10.2a–c). The difference between the current and 2050s shows *Erica* is projected to expand towards the western and northwestern parts of the mountains while maintaining its current range. The difference between the current and 2070s shows *Erica* is projected to continue expanding towards the western and northern parts of the mountains while maintaining a stronghold or showing no change in most of its current range. However, *Erica* will retreat from all lower margins and some parts of the current midaltitude distribution range especially western parents of its current range towards the turn of the century.

**Figure 10:**
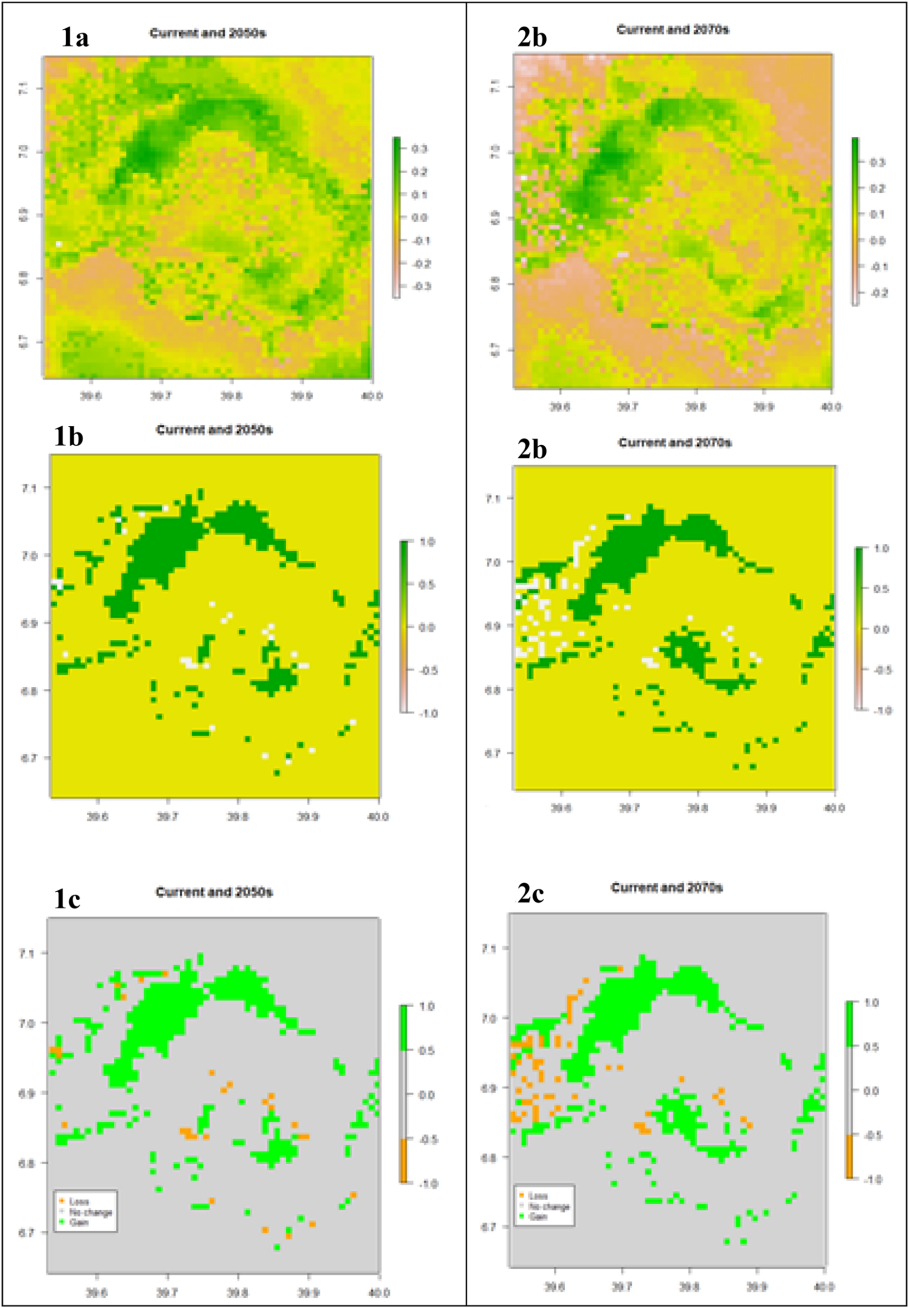
The difference between the averaged models’ ensembles of the three time steps [current and 2050s (Fig. 10.1), and current and 2070s (Fig. 10.2)]. and their respective presence/absence maps (**1b** and **2b** color spectrum). Figure **1c** and **2c** presence/absence maps; a gray area represents absolute absence (unsuitable habitat), green area areas of future gain (gained future highly suitable habitats), while orange area habitat lost (current Erica habitat that will lose suitability in the future) during the span of the time step.

## Discussion

The SDMs and the averaged ensemble models indicated that novel suitable habitats will be created due to climate change across the massive, which will likely be colonized by the ericaceous vegetation. *Erica* is expected to respond to the changes and prevail in the newly created suitable habitat. The different models projected its distribution and provided insight into *Erica's* future habitat such as the area of habitat gain (expansion)—western, northern, and eastern flanks of the massif and afroalpine top; persistence and dominance—most of the midaltitude and its current range; and loss (reduction/ contraction)—most of the lower ranges of current distribution. It provided information about *Erica's* bioclimatic requirements and its current distribution range, including areas that are still remote and inaccessible areas that are free from human habitation.

Tropical alpine and montane ecosystems and the immense biodiversity they harbor are susceptible to climate change induced warming^3,6,14,15^. It is likely that species with a more comprehensive thermal range, such as *Erica*, may track the novel niche and dominate the suitable habitat while receding from the unsuitable habitats. A 1.0 °C increase in mean annual temperature results in a range change of ~ 167 m in altitude but ~ 145 km^−1^ in latitude (based on a temperature lapse rate of − 6.0 °C km^−1^ altitude and − 6.9 °C 1000 km^−1^ latitude)^1,2,16^. The current temperature projections indicate that temperature regimes may shift upward between about 140 and 800 m across afroalpine mountains^14^.

*Erica's* response to climate change will not be different from what has been observed in other places ^1–3^projected an increase in temperature could induce a similar upward shift of altitudinal ecotone succession, leading to a loss of biodiversity at the ecosystem level. Furthermore^71^ and ^4^ indicated that mountain species would respond to climate change by migrating toward higher elevations and summits, in situ resilience of communities and species within microrefugia, adaptation, and evolution through genetic differentiation or extinction. Warren et al.^33^, in their global analysis of the impacts of climatic change on the range of common species, indicated that on average 57 ± 7% of plants are likely to lose ≥ 50% of their current climatic range under RCP8.5.

Climate change is expected to play a significant role in shaping plant communities and displacing ecosystem boundaries along the Bale Mountains massif. As indicated by Steinbauer et al.^72^ in their global assessment, it may result in losses of natural habitats, leading to a rapid loss and extinction of species with their adaptations at lower elevations and mountain top. Furthermore, it may strongly reduce the afroalpine habitat's total area, endangering the many afroalpine habitat specialists and distinctively adapted endemics. The Bale Mountain afroalpine plants may be disadvantaged and outcompeted by *Erica* and similar species under climate change. *Erica* may take advantage and replace many afroalpine endemics with restricted ranges due to its phenotypic plasticity, genetic adaptation to various habitats, high dispersal ability, and broader temperature tolerance. Besides, the movement towards a new climatic niche is a long evolutionary process for many afroalpine specialists^8,13,16,17^. In addition, the unique topography of the plateau does not allow an upward shift because of the smaller area of the few mountain summits. Therefore, the alpine plants are likely to face an ecological dead end^24^.

The predicted *Erica* expansion towards the afroalpine habitat in response to climatic change and the possible effect of these responses on the structure and function of afroalpine ecosystems is essential. Under both future climate change scenarios (RCP4.5 and RCP8.5), many afroalpine plants may decline and suffer local extinction. Overall, global extinction risks increase from 2.8% at present to 5.2% at the international policy target of a 2.0 °C (RCP4.5) post-industrial rise, which is above the^42^ target cumulative emissions of CO_2_ and future non-CO_2_ radiative forcing below or at max 1.5 °C warming. If the Earth warms by 3.0 °C, the extinction risk may rise to 8.5%. One of the current businesses as usual trajectory RCP 8.5 (up to ~ 4.3 °C rise), climate change threatens one in six species (16%) of global species^32^. However, in the Bale Mountains^24^, estimated altitudinal range shifts following a temperature increase of 2.0 °C cause the potential local extinction of 8.7% of all endemic species, and 3.0 °C or 4.0 °C (under RCP8.5) about 36% (of 41 endemic species) local extinction. Plants threatened with extinction include *Sedum mooneyi* M.G. Gilbert, *Anthemis tigreensis* J. Gay ex A.Rich., *Helichrysum harennensis* Mesfin, *Lobelia rhynchopetalum* Hemsl., *Senecio schimperi* Sch.Bip. ex Hochst., *Geranium arabicum* Forssk. subspp. Arabicum, *Carex simensis* Hochst. ex A. Rich., *Helichrysum horridum* Sch. Bip, and *Senecio inornatus* DC.

Along Ethiopian mountains, the alpine and subalpine vegetation have been oscillating in response to the changing climate^27,36,46,47^ and have been shaped by the change in temperature-related bioclimatic variables. Chala et al.^36^, Grabherr et al.^47^ and Ossendorf et al.^73^ indicated that the afroalpine vegetation was expanded towards the lower altitudes during the glaciation periods. McGuire et al.^25^ suggested that the Ericaceous Belt existing today as refugia on high mountains in East Africa would have been more typical of tropical Africa than the present lowland vegetation when the European and African continents came into contact 17 Ma in the mid-Miocene, and the area suitable for exploitation by *Erica* species along the recently uplifted areas would have been more significant than it is today. This is supported by the dispersed distribution of *Erica arborea*.

*Today, Erica* expansion might lead to considerable species replacement, local extinction, and a significant decrease in species richness, especially those endemics across the massif. In many mountain ecosystems, the topography of the steeper slopes may cause small-scale climatic heterogeneity and range of adjacent thermal niches allowing the coexistence of species with differing environmental tolerances in smaller areas^4,74,75^. However, in the Bale Mountains, due to the plateau's relatively flat topography, the role of microrefugia might not be significant. *Erica’s* adaptation to a warming climate and dominance across the landscape will further be aided by gene flow from populations in already warmer areas of the species range [e.g.,^14^].

The Paris Agreement^76^ aims to keep global warming below 2.0 °C while pursuing efforts to limit it to 1.5 °C. IPCC^42^ discusses how the global economy and socio-technical and ecological systems can transition to 1.5 °C consistent pathways and adapt to global warming of 1.5 °C. The impacts of climate change are enormous if global warming exceeds 1.5 °C if the peak temperature is high (e.g., about 2.0 °C). Some impacts of climate change may be long-lasting and/or irreversible, such as the loss of afroalpine ecosystems.

SDMs enable us to build a basic understanding of vascular plants' distribution and diversity patterns in the face of climate change^58,71^. To date, various SDMs based on projection indicate climate impact is inevitable and real. Therefore, adaptation at all levels of human and natural systems is essential. Adaptation and mitigation strategies such as alternative energy sources (biofuels, renewable resources like solar panels, efficient cooking stoves), improving the traditional agricultural systems (extension services, access to credit, changing crop varieties, and adoption of soil and water conservation strategies), local livelihood diversification, good governance, and information on future climate changes are crucial^40,77^.

Besides climate change, species are likely to respond to multiple environmental factors as the environmental conditions change significantly with increasing altitude. Environmental change factors such as geographic barriers that limit dispersal, topography, microrefugia, aspect, and local relief can blur these bioclimatic factors. Therefore, species may not occupy all suitable sites in the future [e.g.,^4,74,75^]. Besides, the effects of orography related precipitation and resource diversity may influence species distribution patterns. Other environmental factors such as atmospheric pressure, CO_2_ concentration, length of the vegetation period, nutrient availability, and soil quality are reduced with altitude. Stressfull events, however, such as the frequency of cold and frosty nights and solar radiation that increase with altitude, might restrict *Erica's* expansion towards the afroalpine plateau.

## Conclusions

The Bale Mountains' ericaceous vegetation will persist as the massif's critical ecosystem even under changing climate. All the models and the ensembled model projected areas of potentially suitable habitats of *Erica* at 1 km resolution and provided *Erica's* possible future distribution range within the Bale massif. We believe our findings will contribute to the scientific basis and understanding of the potential impacts of climate change on the ericaceous vegetation and associate afroalpine flora and other species with high phenotypic plasticity and environmental range. Furthermore, our research that incorporate information from satellite technology, plot data, open source software, and modeling approaches suggests how climate change impacts, and biodiversity conservation management concerns of such remote but highly significant ecosystems could be addressed to improve and support local conservation efforts, set management priorities, and adaptation and mitigation strategies.

Climate change is likely to disrupt and alter the current spatial arrangement, diversity, and distribution of many endemic and non-endemic species of the afroalpine range. In the Bale Mountains, both the upper and lower margins of distributions of *Erica* are highly likely to be affected. Hence, our modeling provided insight into *Erica's* future habitat such as the area of habitat gain (expansion)—western, northern, and eastern flanks of the massif and afroalpine top; persistence and dominance—most of the midaltitude and its current range; and loss (reduction/ contraction)—most of the lower ranges of current distribution. However*, Erica's* future expansion will lead to considerable species replacement, local extinction, and a significant decrease in the species richness of those endemics on the afroalpine plateau. Hence, ecosystems of the afroalpine plateaus and associated unique flora and fauna are highly threatened by climate change.

Climate change adaptation strategies that support the conservation management of the Bale Mountains massif are necessary. Besides, some conservation management and adaptation measures are recommended, such as expanding the park's territory, creating a buffer zone, and limiting human activities and access to the afroalpine region. Overall, there is a need for strict implementation of the existing biodiversity management strategies such as research, monitoring, and periodic assessments of ecosystem status are important.

## Biosketches

Yohannes O Kidane (YOK) is interested in characterizing and protecting biodiversity in tropical landscapes and mountain ecosystems, global change issues, and ecosystems' response to climate change.

Samuel Hoffmann (SH) is a postdoctoral researcher at the Biogeography Department, University of Bayreuth. His research covers biogeography and macroecology, with a special interest in species diversity, climate change, remote sensing, and protected areas.

Mirela Beloiu (MB) is fascinated by forest dynamics and spatial patterns of tree species in mountain areas. Hence, her research focuses on the response of tree species to climate warming and drought.

Anja Jaeschke (AJ) is a postdoctoral researcher at the University of Bayreuth within the Department of Biogeography. Her research focuses on the application and methodological advancement of species distribution models to assess climate change impacts related to nature conservation and vector-borne diseases.

Carl Beierkuhnlein (CB) focuses, among other topics, on the role of biodiversity for ecosystem functioning, on the explanation of spatial patterns of biodiversity, and biogeography in the face of global change.

## Supplementary Information

Supplementary Information.
