# Supplementary material for: Ericaceous vegetation of the Bale Mountains of Ethiopia will prevail in the face of climate change
Source: Sci Rep. 2022 Feb 3;12:1858. doi: 10.1038/s41598-022-05846-z (PMC8813939; doi:10.1038/s41598-022-05846-z)
Supplement: Supplementary file 1 — Supplementary Information. [file 41598_2022_5846_MOESM1_ESM.docx]

**Supplementary materials for the research paper:**

**Ericaceous vegetation of the Bale Mountains of Ethiopia will prevail in the face of climate change**

Yohannes O Kidane*^1^, Samuel Hoffmann^1^, Anja Jaeschke^1^, Mirela Beloiu^1,4^, Carl Beierkuhnlein ^1,2,3^

^1^ Department of Biogeography, Bayreuth, University of Bayreuth, Germany

^2^ Bayreuth Center of Ecology and Environmental Research (BayCEER), Bayreuth, Germany

^3^ Geographical Institute Bayreuth (GIB), Bayreuth, Germany

^4^ Institute of Terrestrial Ecosystems, Department of Environmental System Sciences, ETH Zurich, Zurich, Switzerland

* Corresponding Author

**Attachment I:** R-Script

1. Predictor variables downloading and collinearity test

| # Download environmental predictor variables from Worldclim  bio <- getData("worldclim", var="bio", res=0.5, lon=39, lat=6)  Download the usdam package  library (usdm)  # Test data set for collinearity. We used VIFcor to select the predictor variables those predictors with value above 0.9 will automatically remove from the data set.  V <- vifcor(bio, th=0.9) |
| --- |

1. Data uploading and preprocessing.

| install.packages(c("gdata", "pROC", "Rcpp", "tibble", "repos"))  setwd("C:/----)  library(dismo)  file <- paste(system.file(package="dismo"), "/ex/current/erica.csv", sep="")  file  # read the species occurrence table  erica <- read.table(file, header=TRUE, sep=",")  # read and inspect the values of the file# first rows  head(erica)  # Take the columns latitude and longitude of the occurrence points  erica <- erica[, 2:3]  #select the records that have longitude and latitude data  colnames(erica)  acgeo <- subset(erica, !is.na(lon) & !is.na(lat))  dim(acgeo)  # show some values  acgeo[1:2, c(1:2)]  library(maptools)  data(wrld_simpl)  plot(wrld_simpl, xlim=c(39.58, 40), ylim=c(6.65, 7.1), axes=TRUE, col="skyblue1")  # restore the box around the map  box()  # plot points to visualize the species occuren points  points acgeo$lon, acgeo$lat, col='blue4', pch=21, cex=0.5)  # managing duplicate entries by species  dups2 <- duplicated(acgeo[, c('lon', 'lat')])  # Identify the number of duplicates and removes them from the record  sum(dups2)  [1] 45  # Save the records that are not duplicated  acg <- acgeo[!dups2, ] |
| --- |

1. Cross checking

| #Spatial Points Data Frame was built using the statistical function notation  library(sp)  coordinates(acg) <- ~lon + lat  crs(acg) <- crs(wrld_simpl)  class(acg)  # to use the coordinates to do a spatial query of the polygons in wrldsimpl (a SpatialPolygons DataFrame)  class(wrld_simpl)  ovr <- over(acg, wrld_simpl)  head(ovr)  tail(ovr)  cntr <- ovr$NAME  i <- which(is.na(cntr))  j <- which(cntr != acg$country)  # for the mismatches, bind the country names of the polygons and points  cbind(cntr, acg$country)[j,]  r <- raster(acg)  points(acg[j, ], col='red', pch=20, cex=0.6) |
| --- |

1. Sampling bias

| # create a RasterLayer with the extent of acgeo  r <- raster(acg)  # set the resolution of the cells to (for example) 1 degree  res(r) <- 0.055  # expand (extend) the extent of the RasterLayer a little  r <- extend(r, extent(r)+0.05)  # sample:  acsel <- gridSample(acg, r, n=0.05)  # to illustrate the method and show the result  p <- rasterToPolygons(r)  plot(p, border='skyblue3', legend=T)  points(acg, col='blue', pch=21, cex=0.5)  # selected points in red  points(acsel, cex=1, col='red', pch = 21)  legend(39.825, 7.07, legend=c("presence sampling points", "Subsampling points"),  col=c("blue", "red"), pch=21:21, cex=0.7,  box.lty=1, box.lwd=1, box.col="skyblue3") |
| --- |

| 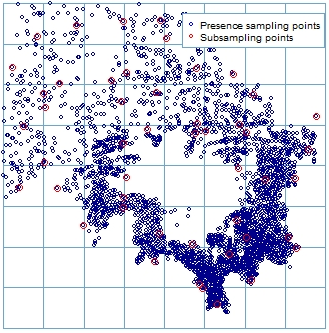 | **Figure 1:** The presence (Navyblue) and subsampling (Red) points. Subsampling uses to reduce sampling biases. It is prepared using the grid sample function, which did a “chess-board” sampling. This is useful to split the data into “training” and “testing” data set records. |
| --- | --- |

1. Absence and background points

| #get the file names  files <- list.files(path=paste(system.file(package="dismo"), '/ex/current/', sep=''), pattern='tif', full.names=TRUE )  #we use the first file to create a RasterLayer  mask <- raster(files[1])  #select 1500 random points set seed to assure that the examples will always have the same random sample.  set.seed(1963)  bg <- randomPoints(mask, 1500 )  # set up the plotting area for two maps  par(mfrow=c(1,2))  plot(!is.na(mask), legend=F)  points(bg, cex=0.5)  #now we repeat the sampling but limit the area of sampling using a spatial extent. The ‘extent’ function further restricts the area from which random locations were drawn.  e <- extent(39.55, 39.99, 6.65, 7.1)  bg2 <- randomPoints(mask, 700, ext=e)  plot(!is.na(mask), legend=F)  plot(e, add=TRUE, col='red')  points(bg2, cex=0.5) |
| --- |

| **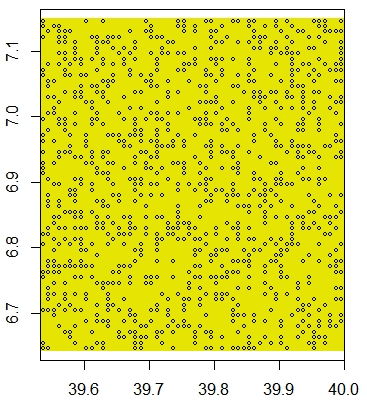(a)** | **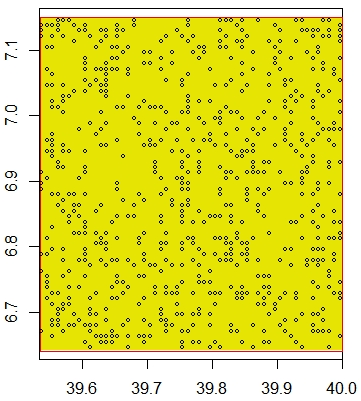(b)** | **Figure 2:** a) Randomly selected 1500 random points set.seed to assure that the examples will always have the same random sample. b) Sampled 700 random background points that were used to characterize and establishes the environmental domain of the study area. |
| --- | --- | --- |

| file <- paste(system.file(package="dismo"), '/ex/current/erica.csv', sep='')  ac <- read.csv(file)  head(ac)  #ac is a data.frame. It must be changed into a Spatial Points Data Frame  coordinates(ac) <- ~lon+lat  projection(ac) <- CRS('+proj=longlat +datum=WGS84')  install.packages("rgeos")  # We create a 'circles' model using an arbitrary radius of 0.5 km  x <- circles(ac, d=500, lonlat=TRUE)  pol <- polygons(x)  # sample randomly from all circles  samp1 <- spsample(pol, 1500, type='random', iter=25) #iter=25  # get unique cells  cells <- cellFromXY(mask, samp1)  length(cells)  cells <- unique(cells)  length(cells)  xy <- xyFromCell(mask, cells)  plot(pol, axes=TRUE)  points(xy, cex=0.5, pch=20, col='blue4')  xy <- xyFromCell(mask, cells)  #Plot to inspect the results:  spxy <- SpatialPoints(xy, proj4string=CRS('+proj=longlat +datum=WGS84'))  o <- over(spxy, geometry(x))  xyInside <- xy[!is.na(o), ]  # extract cell numbers for the circles  v <- extract(mask, x@polygons, cellnumbers=T)  # use rbind to combine the elements in list v  v <- do.call(rbind, v)  # get unique cell numbers from which you could sample  v <- unique(v[,1])  head(v)  # to display the results  m <- mask  m[] <- NA  m[v] <- 1  plot(m, ext=extent(x@polygons)+ 1)  plot(x@polygons, add=T) |
| --- |

| **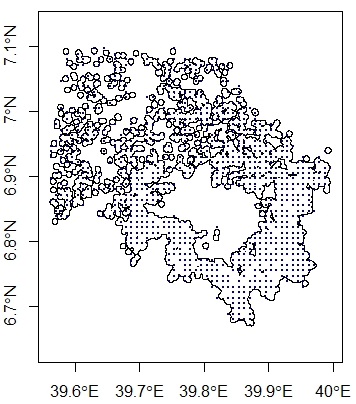(a)** | **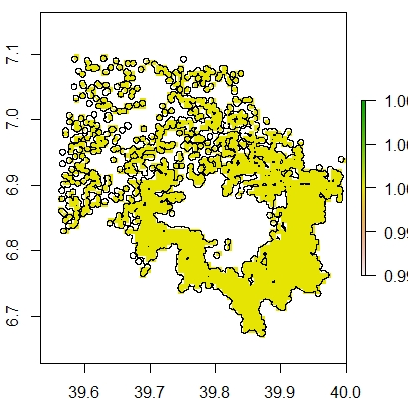 (b)** | **Figure 3:** The species occurrence points were converted into spatial points data frame: a) overlaid on the background points and occurrence circle model of an arbitrary radius of 500m. The circles were ‘dissolved’ using rgeos package, b) The species occurrence points were converted to raster using the raster function. |
| --- | --- | --- |

1. Environmental data entry

| files <- list.files(path=paste(system.file(package="dismo"), '/ex/current/', sep=''), pattern='tif', full.names=TRUE )  files  predictors <- stack(files)  predictors  names(predictors)  plot(predictors)  library(maptools)  data(wrld_simpl)  file <- paste(system.file(package="dismo"), "/ex/current/erica.csv", sep="")  erica <- read.table(file, header=TRUE, sep=',')  plot(predictors, 1)  plot(wrld_simpl, add=TRUE)  # with the points function, "add" is implicit  points(erica, cex=0.5, pch=21, col='blue4') |
| --- |

1. Extracting values from raster

| presvals <- extract(predictors, erica)  #setting random seed to always create the same random set of points for this example  list (predictors)  set.seed(0)  backgr <- randomPoints(predictors, 1500)  absvals <- extract(predictors, backgr)  pb <- c(rep(1, nrow(presvals)), rep(0, nrow(absvals)))  sdmdata <- data.frame(cbind(pb, rbind(presvals, absvals)))  head(sdmdata)  tail(sdmdata)  summary(sdmdata)  #pairs plot of the values of the climate data at the Erica occurrence sites.  pairs(sdmdata[,2:5], cex=0.2, fig=TRUE) |
| --- |

| 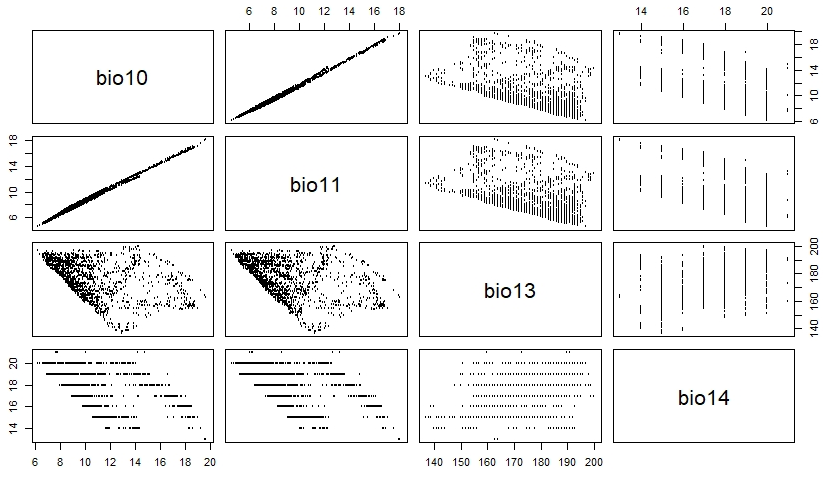 | **Figure 4:** A pairs plot of environmental predictor variables at the Erica occurrence points (sites). The pair plot enables us to visually investigate collinearity in the environmental data (at the presence and background points). |
| --- | --- |

1. Model fitting, prediction, and evaluation
   1. Model fitting

| m <- glm(pb ~ bio11 + bio13 + bio14, data=sdmdata)  class(m)  summary(m)  m2 = glm(pb ~ ., data=sdmdata)  m2  bc <- bioclim(sdmdata[,c('bio11', 'bio13', 'bio14')])  class(bc)  bc  pairs(bc) |
| --- |

| 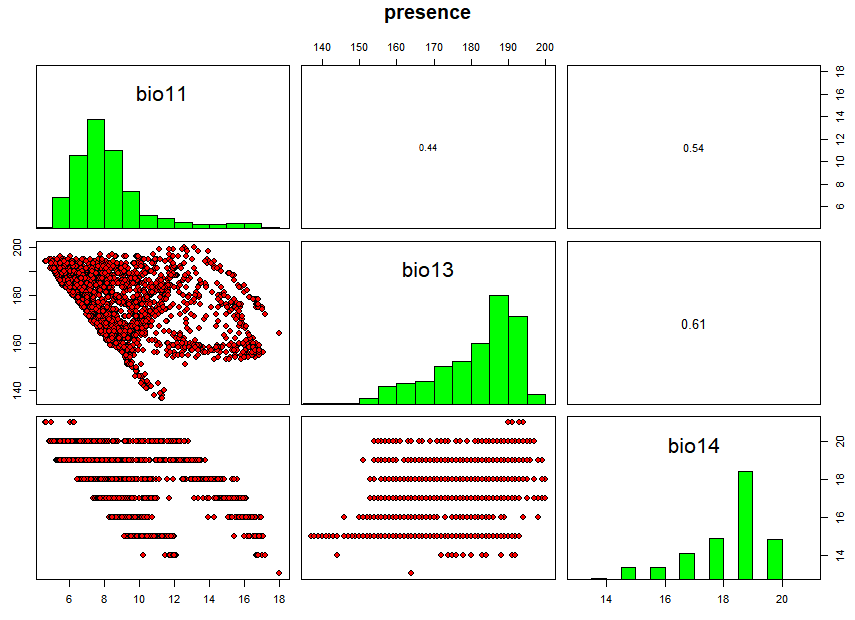 | **Figure 5:** The fitted model which was carried out using only presence value using the ‘prevals’ function. |
| --- | --- |

- 1. Model prediction

| bio11 = c(40, 60, 5)  bio13 = c(150, 115, 15)  bio14 = c(200, 290, 30)  pd = data.frame(cbind(bio11, bio13, bio14))  pd  predict(m, pd)  predict(bc, pd)  response(bc)  names(predictors)  p <- predict(predictors, m)  plot(p) #main="Stacked predictors")  points(erica, cex=0.5, pch=21, col='blue4') |
| --- |

| 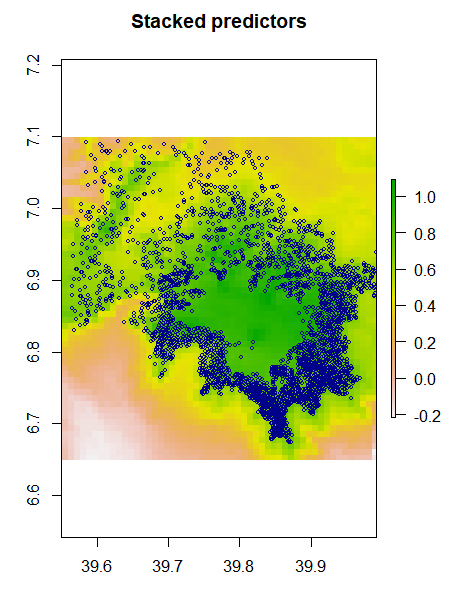 | **Figure 6:**  Layer stacked predictors variables with Erica occurrence point. This is suitability scores map created by the predict function with a Raster object and a model object. |
| --- | --- |

- 1. Model evaluation

| p <- rnorm(700, mean=0.7, sd=0.3)  a <- rnorm(700, mean=0.4, sd=0.4)  par(mfrow=c(1,2))  plot(sort(p), col='red', pch=21)  points(sort(a), col='blue', pch=21)  legend(1, 0.95 * max(a,p), c('precence', 'absence'),  pch=c(21,21), col=c('red', 'blue'))  comb = c(p,a) |
| --- |

| **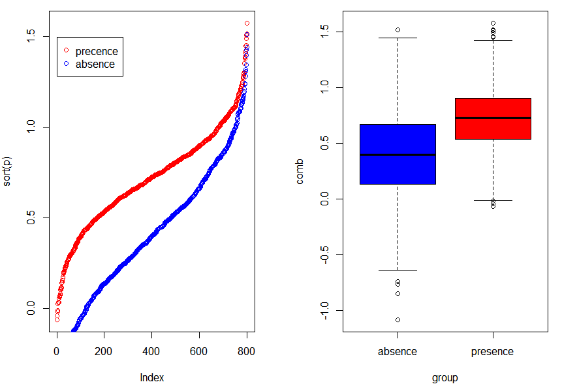(a) (b)** | **Figure 7:** a) Correlation coefficient with two random variables presence((P) has higher values) and represents the predicted value for 700 known locations where the species is present, and absence (a) has lower values, and represents the predicted value for 700 known locations where the species is absent. **b)** box plot of Correlation coefficient. |
| --- | --- |

| samp <- sample(nrow(sdmdata), round(0.5 * nrow(sdmdata)))  traindata <- sdmdata[samp,]  traindata <- traindata[traindata[,1] == 1, 2:11]  testdata <- sdmdata[-samp,]  bc <- bioclim(traindata)  e <- evaluate(testdata[testdata==1,], testdata[testdata==0,], bc)  e  plot(e, 'ROC') |
| --- |

| 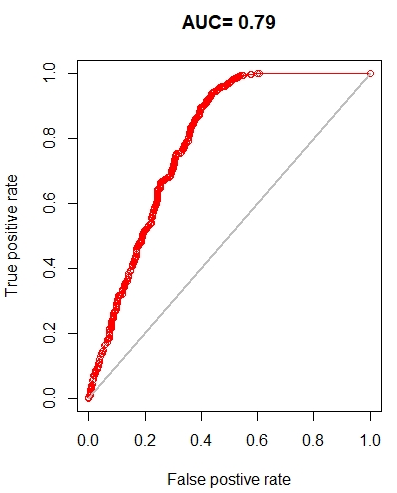 | **Figure 8:** The presence data was divided into two random sets, one for training the models, and one for evaluating the model. The Area Under the Curve (AUC) was calculated. AUC=0.79 indicates that our model has high predicted suitability.  ***NB:*** *In our models the spatial sorting bias of the Area Under the Curve (AUC) was removed. The AUC value used was corrected.* |
| --- | --- |

**NB:** 1. the dismo function K-fold data partitioning was used. It creates a vector that assigns each row in the data matrix to a group (between 1 to k). 2. The spatial sorting bias of the Area Under the Curve (AUC) was removed. The AUC value used was corrected.

| pres <- sdmdata[sdmdata[,1] == 1, 2:11]  back <- sdmdata[sdmdata[,1] == 0, 2:11]  list (pres)  k <- 5  group <- kfold(pres, k)  group[2:11]  unique(group)  e <- list()  for (i in 1:k) {train <- pres[group != i,] test <- pres[group == i,]  bc <- bioclim(train) e[[i]] <- evaluate(p=test, a=back, bc)}  auc <- sapply(e, function(x){slot(x, 'auc')} )  auc  mean(auc)  sapply( e, function(x){x@auc}) |
| --- |

1. Types of algorithms & data used in examples.

| files <- list.files(path(system.file(package="dismo"), '/ex/current/', sep=''), pattern='tif', full.names=TRUE )  predictors <- stack(files)  file <- paste(system.file(package="dismo"), "/ex/current/erica.csv", sep="")  erica <- read.table(file, header=TRUE, sep=',')  erica <- erica [,-1]  presvals <- extract(predictors, erica)  set.seed(0)  backgr <- randomPoints(predictors, 1500)  absvals <- extract(predictors, backgr)  pb <- c(rep(1, nrow(presvals)), rep(0, nrow(absvals)))  sdmdata <- data.frame(cbind(pb, rbind(presvals, absvals)))  pred_nf <- dropLayer(predictors)  group <- kfold(erica, 5)  pres_train <- erica [group!= 1, ]  pres_test <- erica[group == 1, ]  ext = extent(39.55, 39.99, 6.65, 7.1)  backg <- randomPoints(pred_nf, n=1500, ext=ext, extf = 1.25)  colnames(backg) = c('lon', 'lat')  group <- kfold(backg, 5)  backg_train <- backg[group!= 1, ]  backg_test <- backg[group == 1, ]  r = raster(pred_nf, 1)  plot(!is.na(r), col=c('white', 'white4'), legend=F)  colors()  plot(ext, add=TRUE, col='red', lwd=1)  points(backg_train, pch=21, cex=0.4, col='green3')  points(backg_test, pch=21, cex=0.4, col='black')  points(pres_train, pch=21, cex=0.4, col='blue2')  points(pres_test, pch=21,cex=0.4, col='red')  legend(39.58, 6.75, legend=c("Backg train", "Backg test", "Presence train", "Presence test"), col=c("green3", "black", "blue2", "red"), pch=20, cex=0.6, box.lty=1, box.lwd=1, box.col="red") #lty=1, |
| --- |

| 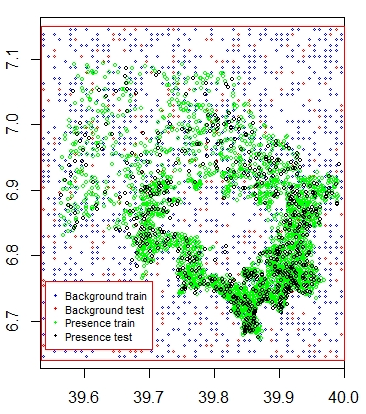 | **Figure 9:** The Erica occurrence points (green) over laid on the stacked environmental variables, from which presence training and testing data sets, and background training and testing data sets were established.  *NB: The random points only occur within the spatial extent of the rasters, and within cells that are not NA, and that there is only a single absence point per cell. Here the background points were restricted to be within 12.5% of the specified extent ‘ext’.* |
| --- | --- |

1. Models
   1. Bioclim

| bc <- bioclim(pred_nf, pres_train)  plot(bc, a=1, b=2, p=0.85)  e <- evaluate(pres_test, backg_test, bc, pred_nf)  tr <- threshold(e, 'spec_sens')  pb <- predict(pred_nf, bc, ext=ext, progress='')  par(mfrow=c(1,2))  plot(pb, main='Bioclim Current')  plot(wrld_simpl, add=TRUE, border='dark gray')  plot(pb > tr, main='presence/absence')  plot(wrld_simpl, add=TRUE, border='dark grey')  points(pres_train, pch= 21, cex=0.6, col='black')  points(backg_train, pch='-', cex=0.5) |
| --- |

| 1. 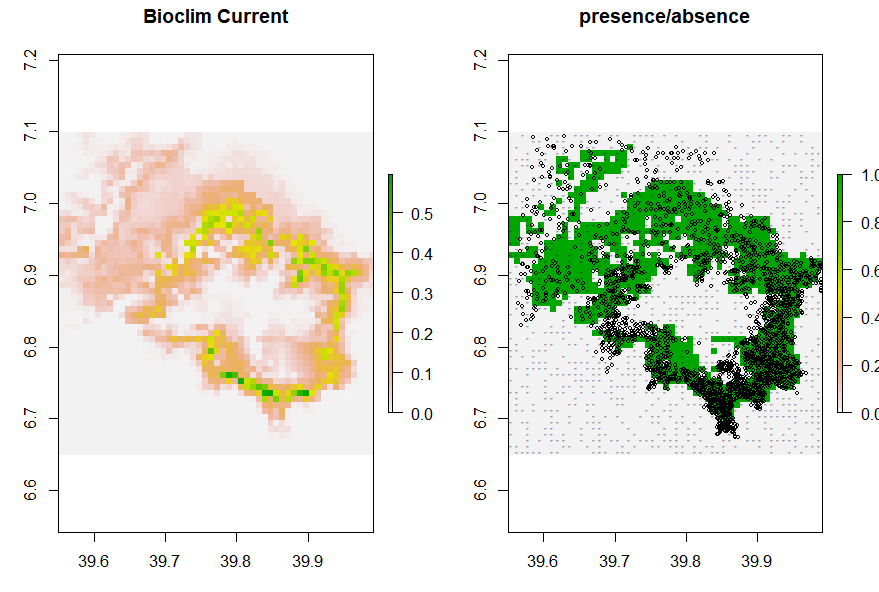 **(b)** | **Figure 10:** a) Bioclim current distribution range suitability scores map.  b) The prediction map that was subjectively classified into  presence (green) /absence (gray) (threshold=0.62). |
| --- | --- |

- 1. Domain

| dm <- domain(pred_nf, pres_train)  ed <- evaluate(pres_test, backg_test, dm, pred_nf)  pd = predict(pred_nf, dm, ext=ext, progress='')  par(mfrow=c(1,2))  plot(pd, main='Domain Current')  plot(wrld_simpl, add=TRUE, border='dark grey')  tr <- threshold(ed, 'spec_sens')  plot(pd > tr, main='presence/absence')  plot(wrld_simpl, add=TRUE, border='dark grey')  points(pres_train, pch= 21, cex=0.6, col='black')  points(backg_train, pch='-', cex=0.5) |
| --- |

| 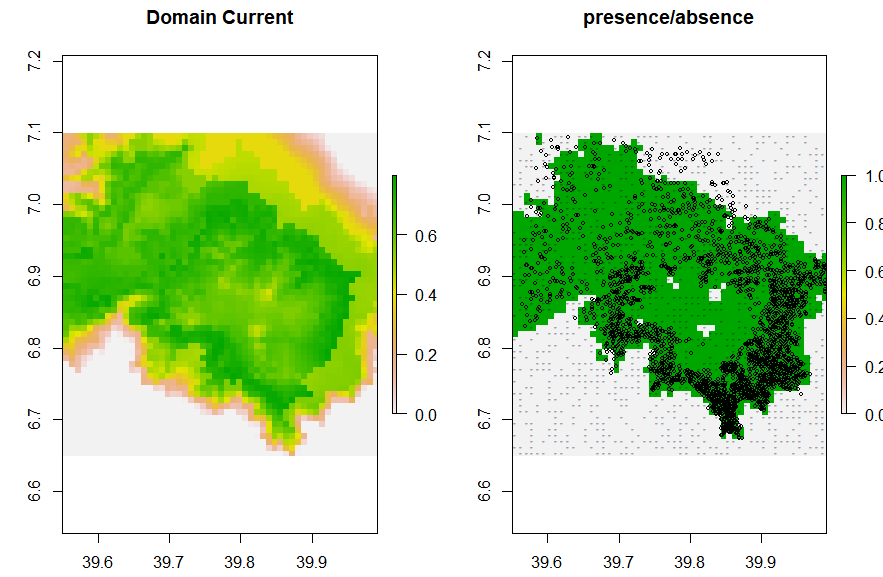 | **Figure 11:** a) Domain **c**urrent distribution range suitability scores map.  b) The prediction map that was subjectively classified into  presence (green) /absence (gray) (threshold=0.51). |
| --- | --- |

- 1. Generalized Linear Models (GLMs).

| train <- rbind(pres_train, backg_train)  pb_train <- c(rep(1, nrow(pres_train)), rep(0, nrow(backg_train)))  envtrain <- extract(predictors, train)  envtrain <- data.frame( cbind(pa=pb_train, envtrain) )  envtrain[,'bioclim'] = factor(envtrain[,'bioclim'], levels=2:11)  head(envtrain)  testpres <- data.frame( extract(predictors, pres_test) )  testbackg <- data.frame( extract(predictors, backg_test) )  family = binomial(link = "logit")  family = gaussian(link = "identity")  family = poisson(link = "log")  gm1 <- glm(pa ~ bio2 + bio8 + bio9 + bio11 + bio13 + bio14 + bio15 + bio18 + bio19, family = binomial(link = "logit"), data=envtrain)  summary(gm1)  coef(gm1)  gm2 <- glm(pa ~ bio2 + bio8 + bio9 + bio11 + bio13 + bio14 + bio15 + bio18 + bio19, family = gaussian(link = "identity"), data=envtrain)  valuate(testpres, testbackg, gm1)  ge2 <- evaluate(testpres, testbackg, gm2)  ge2  pg <- predict(predictors, gm2, ext=ext)  par(mfrow=c(1,2))  plot(pg, main='GLM/gaussian Current')  plot(wrld_simpl, add=TRUE, border='dark grey')  tr <- threshold(ge2, 'spec_sens')  tr  plot(pg > tr, main='presence/absence')  plot(wrld_simpl, add=TRUE, border='dark grey')  points(pres_train, pch= 21, cex=0.6, col='black')  points(backg_train, pch='-', cex=0.5) |
| --- |

| 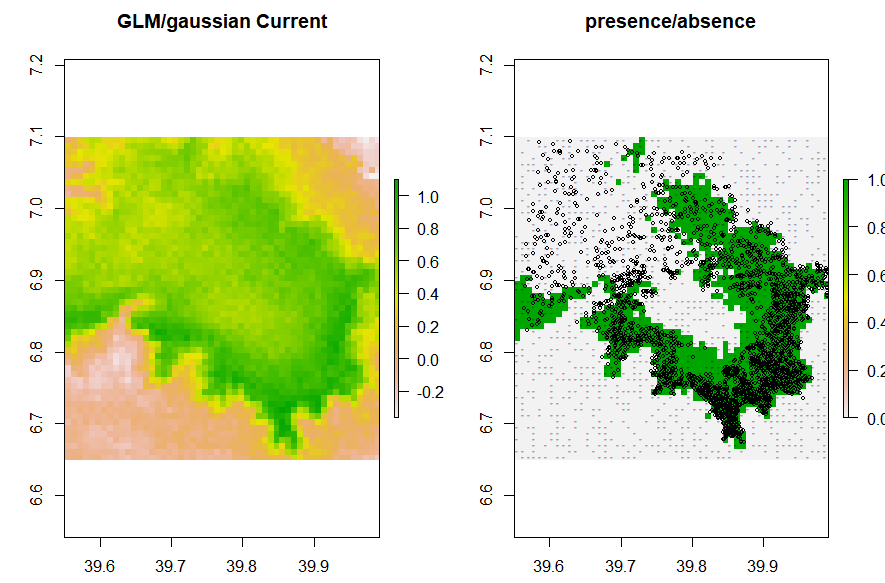 | **Figure 12:** a) GLM/Gaussian current distribution range suitability scores map.  b) The prediction map that was subjectively classified into presence (green) /absence (gray) (threshold= 0.62) |
| --- | --- |

- 1. Support Vector Machines

| library(kernlab)  install.packages('kernlab')  svm <- ksvm(pa ~ bio1 + bio2 + bio3 + bio5 + bio8 + bio9 + bio11 + bio13 + bio14 + bio15 + bio18 + bio19, data=envtrain)  esv <- evaluate(testpres, testbackg, svm)  esv  ps <- predict(predictors, svm, ext=ext)  par(mfrow=c(1,2))  plot(ps, main='SVM Current')  plot(wrld_simpl, add=TRUE, border='dark grey')  tr <- threshold(esv, 'spec_sens')  plot(ps > tr, main='presence/absence')  plot(wrld_simpl, add=TRUE, border='dark grey')  points(pres_train, pch= 21, cex=0.6, col='black')  points(backg_train, pch='-', cex=0.5) |
| --- |

| 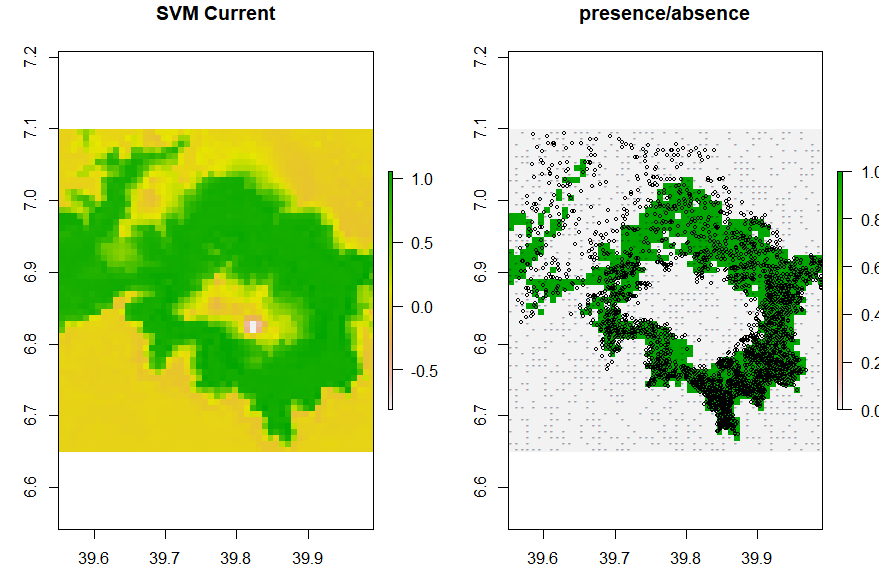 | **Figure 13:** a) SVM current distribution range suitability scores map.  b) The prediction map that was subjectively classified into  presence (green) /absence (gray) (threshold=0.63) |
| --- | --- |

- 1. Combining model predictions

| models <- stack(pb, pd, pg, ps)  names(models) <- c("Bioclim Current", "Domain Current", "GLM Current", "SVM Current")  plot(models)  m <- mean(models)  plot(m, main='Current average score')  auc <- sapply(list(e, ed, ge2, esv), function(x) x@auc)  w <- (auc-0.5)^2  m1 <- weighted.mean( models[[c("pb", "pd", "pg","ps")]], w)  plot(m1, main='weighted mean of four models') |
| --- |

1. Model average scores

| # Weighed mean of all four current models average score  pa <- raster (m)  plot(m)  pa[]<- ifelse(m[] >= 0.3, 1, 0)  plot (pa, main='Weighted mean of Current') |
| --- |

| 1. 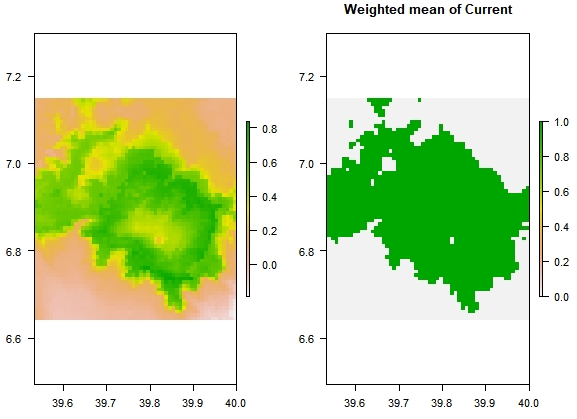 (b) | **Figure 14:** (a) The averaged suitability scores map of all four current models. (b) The averaged prediction map of all four models which was subjectively classified into presence (green) /absence (gray) (threshold = 0.51) |  |  |
| --- | --- | --- | --- |
| # Weighed mean current average score Future 50s  pafa <- raster (mfa)  plot(mfa)  pafa[]<- ifelse(mfa[] >= 0.3, 1, 0)  plot (pafa, main='weighted mean of 2050s') | | |  |
| 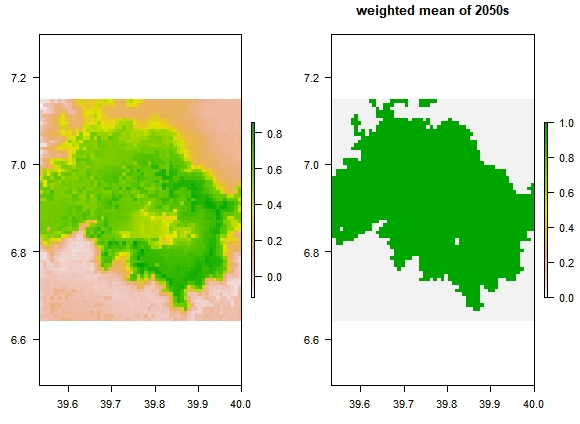 | | | **Figure 15:** (a) The averaged suitability scores map of all four 2050’s models. (b) The averaged prediction map of all four models which was subjectively classified into presence (green) /absence (gray) (threshold = 0.6) |
| # Weighed mean current average score future 70s  pafb <- raster (mfb)  plot(mfb)  pafb[]<- ifelse(mfb[] >= 0.3, 1, 0)  plot (pafb,main='Weighted mean of 2070s') | | |  |

| 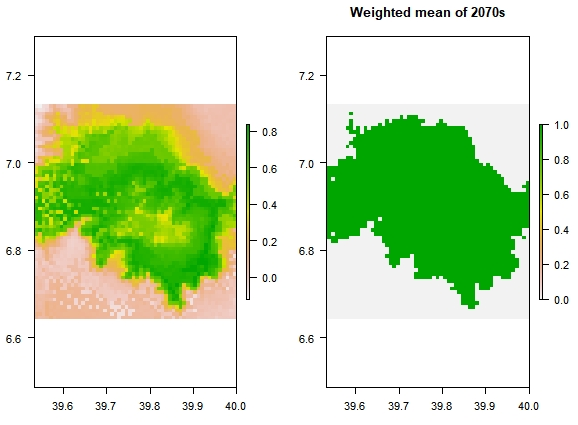 | **Figure 16:** (a) The averaged suitability scores map of all four 2070’s models. (b) The averaged prediction map of all four models which was subjectively classified into presence (green) /absence (gray) (threshold = 0.61) |
| --- | --- |

1. Calculating the difference between models (change Detection)

| # difference between current and future 2050’s  plot(m, main='Current')  m  plot(mfa, main='2050s')  mfa  pac <- mfa-m  plot (pac, main='Current and 2050s')  pac  cl <- colorRampPalette(c('orange', 'lightgray', 'green'))  plot(pac, col=cl(3), main='Current and 2050s') |
| --- |

| 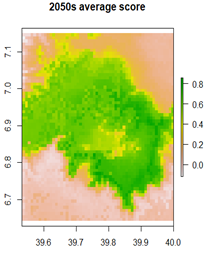 | 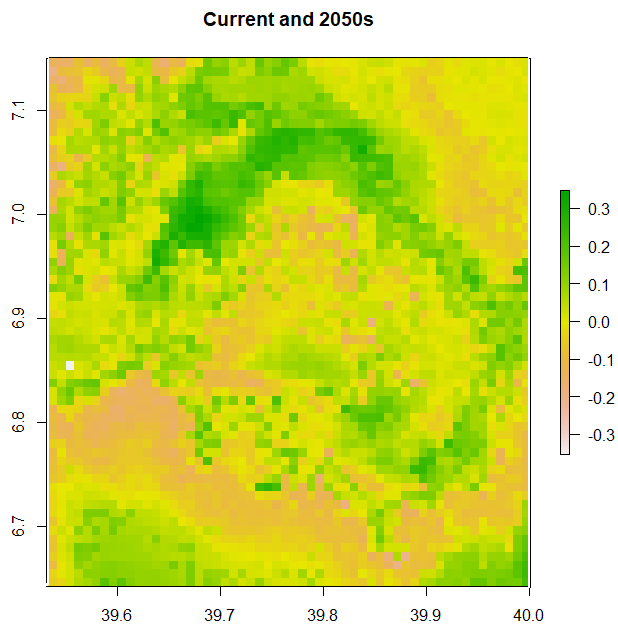 | 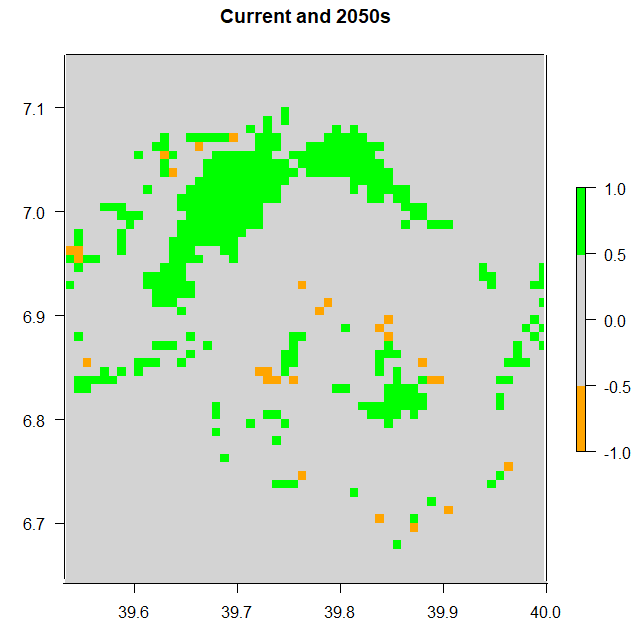 |
| --- | --- | --- |

**Figure 17:** (a) The averaged suitability scores map of the difference between current and 2050’s models. (b) The averaged suitability scores map of the difference between current and 2050’s models which was subjectively classified into areas of gain (Green), loss (Orange) and no change (Gray).

| # difference between current and future 2070’s  plot(m, main='Current')  m  plot(mfb, main='2070s')  mfb  pac <- mfb-m  plot (pac, main='Current and 2070s')  cl <- colorRampPalette(c('orange', 'lightgray', 'green'))  plot(pac, col=cl(3), main='Current and 2070s') |
| --- |

| 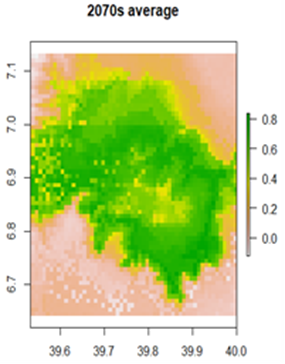 | 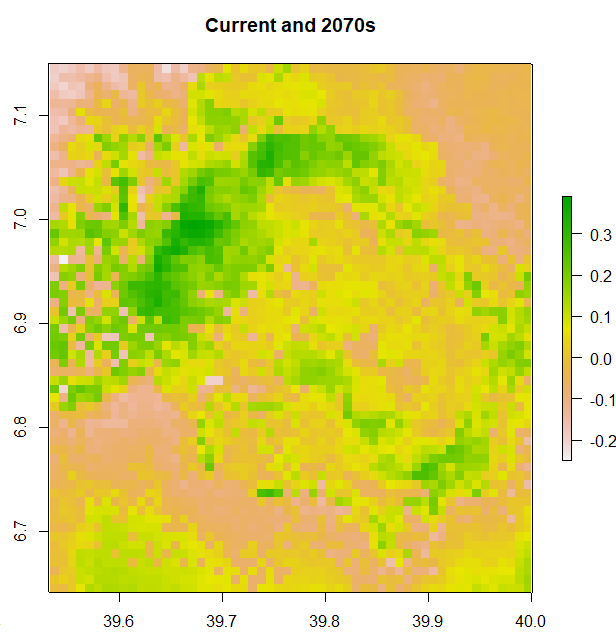 | 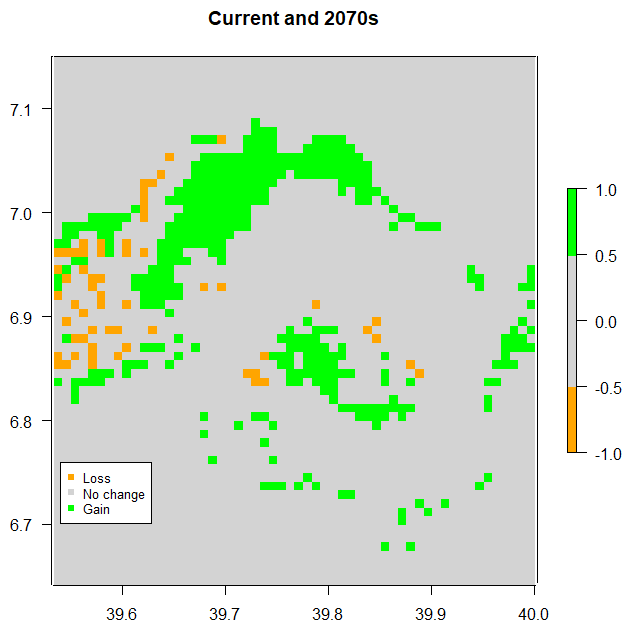 |
| --- | --- | --- |

**Figure 18:** (a) The averaged suitability scores map of the difference between current and 2070’s models. (b) The averaged suitability scores map of the difference between current and 2070’s models which was subjectively classified into areas of gain (Green), loss (Orange) and no change (Gray).

1. **Reference**

Hijmans, R.J., Cameron, S.E., Parra, J.L., Jones, P.G., and Jarvis, A., 2005. Very high-resolution interpolated climate surfaces for global land areas. International Journal of Climatology. 25, 1965-1978.

Hijmans, R.J., van Etten, J., 2011. raster: Geographic Analysis and Modeling with Raster Data. R package version 1.8-39, URL <http://CRAN.R-project.org/package=raster> (accessed July 2020).

Hijmans, R.J., and Elith, J., 2017. Species distribution modeling with R. [https://cran.r-project.org/web/ packages/dismo/vignettes/sdm.pdf](https://cran.r-project.org/web/%20packages/dismo/vignettes/sdm.pdf) (accessed April 2021).

Naimi, B., and Araújo, M.B., 2016. SDM: a reproducible and extensible R platform for species distribution modeling. Ecography. 39, 368–375. DOI: 10.1111/ecog.01881.

Naimi, B., Hamm, N.A.S., Groen, T.A., Skidmore, A.K., and Toxopeus, A.G., 2014. Where is positional uncertainty a problem for species distribution modeling? Ecography. 37 (2), 191-203.

R Core Team. 2019. R: A language and environment for statistical computing. R Foundation for Statistical Computing [Internet]. [http://www.R-project.org/](http://www.r-project.org/).

**Attachment II:** Erica sampling data points

| N^O^ | species | lat | lon | alt |
| --- | --- | --- | --- | --- |
| 1 | Erica | 6.712718 | 39.90395 | 3200 |
| 2 | Erica | 6.804442 | 39.95387 | 3200 |
| 3 | Erica | 6.762687 | 39.96884 | 3201 |
| 4 | Erica | 6.923988 | 39.97547 | 3202 |
| 5 | Erica | 6.816775 | 39.67908 | 3202 |
| 6 | Erica | 6.855978 | 39.96332 | 3202 |
| 7 | Erica | 6.814859 | 39.6754 | 3203 |
| 8 | Erica | 6.867394 | 39.66312 | 3203 |
| 9 | Erica | 6.982194 | 39.91281 | 3203 |
| 10 | Erica | 6.730306 | 39.8253 | 3204 |
| 11 | Erica | 6.830132 | 39.56829 | 3205 |
| 12 | Erica | 6.841292 | 39.58601 | 3205 |
| 13 | Erica | 6.883203 | 39.97051 | 3206 |
| 14 | Erica | 6.740665 | 39.94003 | 3206 |
| 15 | Erica | 6.711997 | 39.83326 | 3206 |
| . |  |  |  |  |
| . |  |  |  |  |
| 3219 | Erica | 6.800517 | 39.90305 | 3884 |
| 3220 | Erica | 6.793763 | 39.8877 | 3884 |

**Table 1:** The Erica occurrence points with latitude, longitude, and altitude of occurrence.
